# Antibiotics for common infections in primary care before, during and after the COVID-19 pandemic: cohort study of extent of prescribing based on risks of infection-related hospital admissions

**DOI:** 10.1177/01410768251328997

**Published:** 2025-04-03

**Authors:** Ali Fahmi, Ya-Ting Yang, Xiaomin Zhong, Alexander Pate, Anita Sharma, Simon Watts, Darren M Ashcroft, Ben Goldacre, Brian MacKenna, Jon Massey, Amir Mehrkar, Seb Bacon, Brian McMillan, Paul Dark, Kieran Hand, Victoria Palin, Tjeerd Pieter van Staa

**Affiliations:** 1 Centre for Health Informatics, School of Health Sciences, Faculty of Biology, Medicine, and Health, The University of Manchester, Manchester M13 9PL, UK; 2 Chadderton South Health Centre, Eaves Lane, Chadderton, Oldham, UK; 3 North West Ambulance Service, Manchester NHS Foundation Trust, Manchester M45 6AQ, UK; 4 Centre for Pharmacoepidemiology and Drug Safety, School of Health Sciences, Faculty of Biology, Medicine and Health, The University of Manchester, Manchester M13 9PL, UK; 5 NIHR Greater Manchester Patient Safety Translational Research Centre, School of Health Sciences, Faculty of Biology, Medicine and Health, The University of Manchester, Manchester M13 9PL, UK; 6 Bennett Institute for Applied Data Science, Nuffield Department of Primary Care Health Sciences, University of Oxford, Oxford OX2 6GG, UK; 7 Centre for Primary Care and Health Services Research, Faculty of Biology, Medicine and Health, The University of Manchester, Manchester, UK; 8 Division of Immunology, Immunity to Infection and Respiratory Medicine, The University of Manchester, Manchester, UK; 9 NHS England, Wellington House, London SE1 8UG, UK; 10 Maternal and Foetal Health Research Centre, Division of Developmental Biology and Medicine, The University of Manchester, Manchester M13 9WL, UK

**Keywords:** Antibiotics, infections, hospital admissions, antimicrobial resistance, COVID-19 pandemic

## Abstract

**Objectives:**

Antibiotics are effective in treating bacterial infections, but they carry the risks of antimicrobial resistance and effectiveness loss. This study aimed to assess whether antibiotics for common infections are prescribed in a risk-based manner and how this changed during the COVID-19 pandemic.

**Design:**

Cohort study of common infections and antibiotic prescribing.

**Setting:**

With the approval of NHS England, we accessed pseudonymised patient-level electronic health records of primary care data from The Phoenix Partnership through OpenSAFELY.

**Participants:**

We included adults registered at general practices in England with a record of common infection, including lower respiratory tract infection (LRTI), upper respiratory tract infections (URTI) and lower urinary tract infection (UTI), from January 2019 to March 2023. Patients with a record of COVID-19 were excluded.

**Main outcome measures:**

Patient-specific risks of infection-related hospital admission were estimated for each infection using risk prediction scores for patients who were not prescribed an antibiotic. The infection cohorts were then grouped into risk deciles, and probabilities of being prescribed an antibiotic were assessed.

**Results:**

We found 15,719,750 diagnoses of common infections. Of them, 450,215 (2.86%) were hospitalised in the 30 days after the diagnosis and 10,429,060 (66.34%) were prescribed an antibiotic. There were substantial differences in observed rates of hospital admissions between the lowest and highest risk deciles (25-fold difference in URTI). The probability of being prescribed an antibiotic for LRTI or UTI was unrelated to hospital admission risk, and that for URTI was weakly related to hospital admission risk. During the COVID-19 pandemic, the level of risk-based antibiotic prescribing reduced.

**Conclusions:**

There is a need to better target antibiotics in primary care to patients with worse prognosis and strengthen treatment guidelines in personalisation of prescribing.

## Introduction

Antibiotics have been revolutionary in the history of healthcare for treating bacterial infections. Yet in the 21st century, antimicrobial resistance (AMR) has been recognised as one of the biggest threats to global public health.^1^ A World Health Organization report on antimicrobial surveillance has shown a worrying level of AMR globally.^2^ With AMR projected to become a greater threat than cancer by 2050, many policies have been put in place to improve prescribing of antibiotics.^3^ The major policy focus has been on decreasing overall levels of antibiotic prescribing and improving the appropriateness of prescribing.^4^ Yet these strategies alone are unlikely to solve such a substantial global health burden.^5^

Primary care in England accounted for 80.5% of all prescribed antibiotics in 2021 and has witnessed a 17.2% reduction in antibiotic use between 2017 and 2021.^6^ England’s National Institute for Health and Care Excellence has disseminated guidelines for antimicrobial stewardship. These guidelines highlight the necessity of taking into account the risk of AMR and recommend considering risk of complications but provide no specific advice on how to consider these risks. Prognosis (i.e. risk of disease complications) is generally a key consideration for clinicians assessing possible interventions, with the exception of symptomatic treatments (such as painkillers).

The aims of this study were to assess whether antibiotics for common infections in primary care are prescribed according to a patient’s prognosis (i.e. in a risk-based manner based on risks of infection-related complications), and how the COVID-19 pandemic impacted any risk-based prescribing of antibiotic.

## Methods

We used the OpenSAFELY platform (https://opensafely.org/) that contains pseudonymised data of electronic health records (EHRs). OpenSAFELY provides access to individual-level primary care data of almost 24 million people from The Phoenix Partnership (TPP). OpenSAFELY–TPP data are linked to external databases, such as Hospital Episode Statistics.

## Study population

We included people aged 18 years or older registered with a general practice who had a record of diagnosis of common infections from January 2019 to March 2023 and excluded those with a record of COVID-19 diagnosis 90 days before to 30 days after the diagnosis of common infections. The reason for this exclusion was to eliminate viral COVID-19 infections and focus on infections that are frequently bacterial and treated with antibiotics. We created cohorts for common infections, namely lower respiratory tract infection (LRTI), upper respiratory tract infections (URTI, including specific URTI, cough, cold with cough and sore throat), lower urinary tract infection (UTI), sinusitis, otitis externa and otitis media. These cohorts were updated versions of the cohorts of a previous study,^7^ with a longer period (January 2019 to March 2023 instead of January 2019 to August 2022) and therefore larger cohorts. Similar to this previous study,^7^ we stratified the cohorts into incident or prevalent and whether antibiotics were prescribed within five days period after the infection diagnosis was recorded. Incident infections were defined as those that were not previously recorded during the 42-day period before the infection diagnosis, and prevalent infections were those that were not incident. Twenty different infection diagnoses for each common infection were extracted to create the cohorts for common infections. We used the codes of the 10th revision of International Classification of Diseases to identify infection-related hospital admissions.^8^ COVID-19 diagnosis records were extracted from two main sources: the UK Health Security Agency Second Generation Surveillance System (SGSS)^9^ and general practitioner (GP) records of COVID-19 diagnosis in primary care.

## Study variables

We extracted variables that were potentially associated with risks of infection-related hospital admissions^10^ and were recorded in the EHRs, including age, sex, Charlson co-morbidity index (CCI),^11^ body mass index (BMI), flu vaccination in the one year before, region of England, socioeconomic level measured by the Index of Multiple Deprivation (IMD), ethnicity, smoking status, season of the year and history of antibiotics measured by the count of prescribed antibiotics in the one year before.

## Statistical analyses

We adopted four analysis steps, as described in detail below (and shown in the flowchart of analyses in Supplementary Figure S1):

Step 1 – We developed and validated Cox proportional-hazards models in the cohorts not prescribed antibiotic. Predictors of these models included age, sex, CCI, BMI, flu vaccination, region, IMD, ethnicity, smoking status, season and history of antibiotics, as described in the previous study.^7^ These models predicted risks of infection-related hospital admission in a 30-day follow-up period after the common infection record. We randomly split each infection cohort into development (75%) and validation (25%) sets, and we assigned missingness indicator to handle missing values.
Step 2 – The patient-specific predictions were then applied to the users of antibiotic, estimating their risks of infection-related hospital admissions if they had not been treated with an antibiotic. We also applied these patient-specific risk predictions to the non-users of antibiotic.
Step 3 – We combined cohorts of antibiotic users and non-users and grouped the predicted risks of infection-related hospital admissions into 10 deciles.
Step 4 – We conducted two sets of analyses. In the first set of analyses, we compared the risk deciles with the records of prescribed antibiotics to predict the chance of being prescribed antibiotic for common infections – that is, the outcome of interest was the probability of being prescribed antibiotics for common infections. For this, we used logistic regression models to predict the probability of antibiotic prescribing using the 2nd to 10th risk deciles as covariates. In the second set of analyses, we used logistic regression models to assess the effect of individual risk factors on the probability of being prescribed an antibiotic. Risk factors (i.e. covariates) of these analyses included sex, age, BMI, ethnicity, CCI, smoking status, IMD, season, region, flu vaccination and antibiotic history. There seem to be an extra space between this paragraph and the next one.

In addition, we stratified the cohorts by the status of COVID-19 into four periods: (1) pre-pandemic (January 2019 to December 2019); (2) beginning of the pandemic (January 2020 to April 2020); (3) during the pandemic (May 2020 to April 2021); and (4) after the second national lockdown (May 2021 to March 2023). We dropped the second period from the analysis because of varying changes in behaviour of the population and treatment pathways for common infections in primary care.^12^ For the remaining three periods, we replicated the analyses of Steps 1–4.

The performance of the logistic regression models was measured with the area under the receiver operating characteristic (AUROC). Jupyter Notebooks with Python 3.10.4 were used for the analyses. The lifelines package version 0.27.0 was used for the Cox models and the scikit-learn package version 1.0.2 for the logistic regression models. The notebooks are available in the risk-based antibiotic prescribing branch of a GitHub repository (https://github.com/opensafely/amr- uom-brit/tree/ab_rx_risk_based).

## Results

We found a total of 15,719,750 diagnoses of common infections from January 2019 to March 2023, of which 14,151,895 (90.03%) were incident and 1,567,855 (9.97%) were prevalent. Of incident common infections, 9,464,005 (66.87%) were prescribed antibiotics (86.76% for incident LRTI, 57.69% for URTI and 86.62% for UTI). Table 1 shows the main baseline characteristics of the cohorts of incident and prevalent common infections, namely LRTI, URTI and UTI, with and without prescribed antibiotics (all counts are rounded). Supplementary Tables S1–S3 show additional baseline characteristics that generally showed only small differences between users and non-users of antibiotics in characteristics such as age and CCI.

**Table 1.** Baseline characteristics of the cohorts of common infections including lower respiratory tract infection (LRTI), upper respiratory tract infection (URTI) and urinary tract infection (UTI), without prescribed antibiotics, using data from January 2019 to March 2023.

	LRTI				URTI				UTI			
	Incident		Prevalent		Incident		Prevalent		Incident		Prevalent	
	No ABs^a^	With ABs	No ABs	With ABs	No ABs	With ABs	No ABs	With ABs	No ABs	With ABs	No ABs	With ABs
Total, *N* cases	318,780	2,089,600	73,755	202,915	3,063,345	4,177,380	340,830	369,325	351,150	2,273,825	91,810	312,800
Age, *N* (%)												
18–24	13,300 (4.17)	84,885 (4.06)	1755 (2.38)	3900 (1.92)	190,845 (6.23)	318,190 (7.62)	18,810 (5.52)	19,330 (5.23)	30,190 (8.60)	150,970 (6.64)	5,165 (5.63)	13,750 (4.40)
25–34	26,665 (8.36)	201,035 (9.62)	4590 (6.22)	12,080 (5.95)	320,890 (10.48)	568,525 (13.61)	32,815 (9.63)	35,365 (9.58)	41,610 (11.85)	255,690 (11.24)	7595 (8.27)	22,840 (7.30)
35–44	29,150 (9.14)	242,435 (11.60)	6310 (8.55)	18,095 (8.92)	322,010 (10.51)	547,970 (13.12)	35,730 (10.48)	38,355 (10.39)	33,930 (9.66)	245,185 (10.78)	6,790 (7.40)	23,775 (7.60)
45–54	40,375 (12.67)	338,350 (16.19)	9655 (13.09)	29,485 (14.53)	449,360 (14.67)	655,615 (15.69)	52,615 (15.44)	54,175 (14.67)	41,165 (11.72)	308,160 (13.55)	9250 (10.08)	35,115 (11.23)
55–64	49,585 (15.56)	396,800 (18.99)	12,155 (16.48)	39,040 (19.24)	579,450 (18.92)	726,060 (17.38)	65,230 (19.14)	67,960 (18.40)	44,595 (12.70)	338,860 (14.90)	12,320 (13.42)	46,360 (14.82)
65–74	63,300 (19.86)	401,845 (19.23)	14,860 (20.15)	45,155 (22.25)	653,135 (21.32)	700,685 (16.77)	68,575 (20.12)	74,395 (20.14)	58,570 (16.68)	430,715 (18.94)	18,755 (20.43)	71,335 (22.80)
75+	96,405 (30.24)	424,250 (20.30)	24,435 (33.13)	55,165 (27.19)	547,640 (17.88)	660,330 (15.81)	67,075 (19.68)	79,735 (21.59)	101,090 (28.79)	544,240 (23.94)	31,935 (34.78)	99,625 (31.85)
Sex, *N* (%)												
Female	182,340 (57.20)	1,266,015 (60.59)	44,600 (60.47)	125,110 (61.66)	1,735,255 (56.65)	2,606,720 (62.40)	202,770 (59.49)	231,510 (62.68)	250,870 (71.44)	1,880,855 (82.72)	65,405 (71.24)	250,080 (79.95)
Male	136,440 (42.80)	823,585 (39.41)	29,155 (39.53)	77,805 (38.34)	1,328,090 (43.35)	1,570,660 (37.60)	138,060 (40.51)	137,815 (37.32)	100,280 (28.56)	392,970 (17.28)	26,405 (28.76)	62,720 (20.05)
Ethnicity, *N* (%)												
White	269,570 (84.56)	1,666,090 (79.73)	63,525 (86.12)	173,350 (85.43)	2,409,865 (78.67)	3,222,300 (77.14)	274,160 (80.44)	305,145 (82.62)	293,560 (83.60)	1,857,440 (81.69)	79,565 (86.66)	271,170 (86.69)
Asian	14,445 (4.53)	114,990 (5.50)	2,925 (3.96)	8,735 (4.31)	144,655 (4.72)	265,790 (6.36)	16,720 (4.91)	18,580 (5.03)	15,955 (4.54)	102,975 (4.53)	3,160 (3.44)	10,900 (3.48)
Black	3875 (1.21)	21,155 (1.01)	600 (0.81)	1305 (0.64)	37,805 (1.23)	51,860 (1.24)	4345 (1.27)	3360 (0.91)	4430 (1.26)	23,550 (1.04)	715 (0.78)	1945 (0.62)
Mixed	2535 (0.79)	16,330 (0.78)	505 (0.68)	1390 (0.69)	24,420 (0.80)	36,935 (0.88)	2745 (0.80)	2770 (0.75)	3355 (0.95)	18,935 (0.83)	695 (0.76)	2325 (0.74)
Other	4230 (1.33)	23,830 (1.14)	710 (0.96)	1740 (0.86)	43,725 (1.43)	58,120 (1.39)	4895 (1.43)	4435 (1.20)	5220 (1.49)	25,880 (1.14)	1050 (1.14)	2575 (0.82)
Unknown	24,125 (7.57)	247,205 (11.83)	5500 (7.45)	16,395 (8.08)	402,865 (13.15)	542,375 (12.98)	37,985 (11.14)	35,035 (9.49)	28,635 (8.15)	245,045 (10.78)	6630 (7.22)	23,890 (7.64)
CCI,^b^ *N* (%)												
Very low (=0)	156,375 (49.05)	1,135,610 (54.35)	32,890 (44.59)	92,085 (45.38)	1,788,140 (58.37)	2,468,800 (59.10)	191,250 (56.11)	187,595 (50.79)	209,195 (59.57)	1,428,850 (62.84)	49,625 (54.05)	175,130 (55.99)
Low (=1 and =2)	118,970 (37.32)	747,880 (35.79)	29,405 (39.87)	82,985 (40.90)	1,002,915 (32.74)	1,360,160 (32.56)	113,920 (33.42)	138,015 (37.37)	101,445 (28.89)	640,095 (28.15)	29,160 (31.76)	98,960 (31.64)
Medium (=3 and =4)	33,045 (10.37)	164,240 (7.86)	8645 (11.72)	21,835 (10.76)	218,020 (7.12)	278,550 (6.67)	27,835 (8.17)	34,225 (9.27)	30,665 (8.73)	162,295 (7.14)	9715 (10.58)	29,955 (9.58)
High (=5 and =6)	7695 (2.41)	32,235 (1.54)	2085 (2.82)	4565 (2.25)	42,435 (1.39)	53,810 (1.29)	5945 (1.75)	7305 (1.98)	7430 (2.12)	33,185 (1.46)	2485 (2.70)	6630 (2.12)
Very high (≥7)	2690 (0.84)	9635 (0.46)	735 (1.00)	1445 (0.71)	11,835 (0.39)	16,050 (0.38)	1885 (0.55)	2185 (0.59)	2415 (0.69)	9400 (0.41)	830 (0.90)	2125 (0.68)
Flu vaccination, *N* (%)												
Yes	166,700 (52.29)	987,520 (47.26)	41,650 (56.47)	114,920 (56.63)	1,458,165 (47.60)	2,437,180 (58.34)	178,610 (52.40)	181,855 (49.24)	158,790 (45.22)	1,028,465 (45.23)	49,065 (53.44)	171,320 (54.77)
No	152,075 (47.71)	1,102,080 (52.74)	32,105 (43.53)	88,000 (43.37)	1,605,180 (52.40)	1,740,195 (41.66)	162,220 (47.60)	187,475 (50.76)	192,360 (54.78)	1,245,355 (54.77)	42,745 (46.56)	141,485 (45.23)
Period												
Pre-pandemic	104,330 (32.73)	772,975 (36.99)	30,780 (41.73)	86,885 (42.82)	1,005,590 (32.83)	1,427,135 (34.16)	119,710 (35.12)	138,685 (37.55)	92,800 (26.43)	605,420 (26.63)	26,640 (29.02)	87,830 (28.08)
During pandemic	38,930 (12.21)	144,855 (6.93)	7010 (9.50)	13,340 (6.57)	427,470 (13.95)	368,475 (8.82)	47,825 (14.03)	33,750 (9.14)	73,480 (20.92)	520,155 (22.88)	21,400 (23.31)	72,170 (23.07)
After 2nd lockdown	143,035 (44.87)	957,840 (45.84)	26,470 (35.89)	75,910 (37.41)	1,407,810 (45.96)	2,032,405 (48.65)	141,585 (41.54)	155,885 (42.21)	159,635 (45.46)	959,900 (42.22)	36,770 (40.05)	126,255 (40.36)
Count of antibiotic prescription, mean (SD^c^)	2.16 (2.82)	2.7 (2.37)	3.36 (2.92)	4.49 (2.74)	1.44 (3.37)	2.52 (2.48)	2.39 (2.89)	3.92 (2.27)	2.3 (3.09)	3.22 (2.90)	4.06 (3.50)	5.51 (3.46)

The cohort consists of incident infections with no prescribed antibiotics, incident infections with prescribed antibiotics, prevalent infections with no prescribed antibiotics and prevalent infections with prescribed antibiotics.

a ABs: antibiotics prescribed or not.

b CCI, Charlson Comorbidities Index, measured from 17 weighted conditions, including myocardial infarction, congestive heart failure, peripheral vascular disease, cerebrovascular disease, dementia, chronic pulmonary disease, connective tissue disease, ulcer disease, mild liver disease, diabetes, hemiplegia, moderate or severe renal disease, diabetes with complications, any malignancy (including leukaemia and lymphoma), moderate or severe liver disease, metastatic solid tumour and AIDS.

c SD: standard deviation.

Table 2 shows the counts and observed rates of infection-related hospital admission cases in deciles of predicted probability of hospital admission for incident LRTI, URTI and UTI combining users and non-users of antibiotics (all counts are rounded). The observed rates of hospital admissions were strongly related to the predicted risk (for URTI, 25-fold difference in risk between lowest and highest decile). Supplementary Tables S4–S6 show these results for prevalent and other infections.

**Table 2.** Count and observed rate of infection-related hospital admission in deciles of predicted probability of hospital admission related to incident common infections, including lower respiratory tract infection (LRTI), upper respiratory tract infection (URTI) and urinary tract infection (UTI), using data from January 2019 to March 2023.

Deciles^a^ of predicted risk	LRTI*N* cases(observed rate^b^ of hospital admissions)	URTI*N* cases(observed rate of hospital admissions)	UTIN cases(observed rate of hospital admissions)
Decile 1 (lowest)	1030 (4.3)	1465 (2.0)	945 (3.6)
Decile 2	2100 (8.7)	4545 (6.1)	1845 (7.0)
Decile 3	2650 (11.0)	5665 (7.6)	2440 (9.3)
Decile 4	3020 (12.5)	6300 (8.5)	2820 (10.8)
Decile 5	3725 (15.5)	7155 (9.6)	3605 (13.7)
Decile 6	4690 (19.5)	8350 (11.2)	5090 (19.4)
Decile 7	6225 (25.8)	9735 (13.1)	7490 (28.5)
Decile 8	9065 (37.6)	13,600 (18.3)	11,280 (43.0)
Decile 9	13,185 (54.7)	21,940 (29.5)	15,740 (60.0)
Decile 10 (highest)	17,995 (74.7)	38,270 (51.4)	23,435 (89.3)

a Deciles are calculated by grouping predicted probability of infection-related hospital admission using Cox models.

b Rate is the number of cases per 1000 patients with common infection, calculated by dividing the count of infection-related hospital admission cases (numerator) by the count of infection diagnosis (denominator) and then multiplied by 1000.

**Figure 1.**
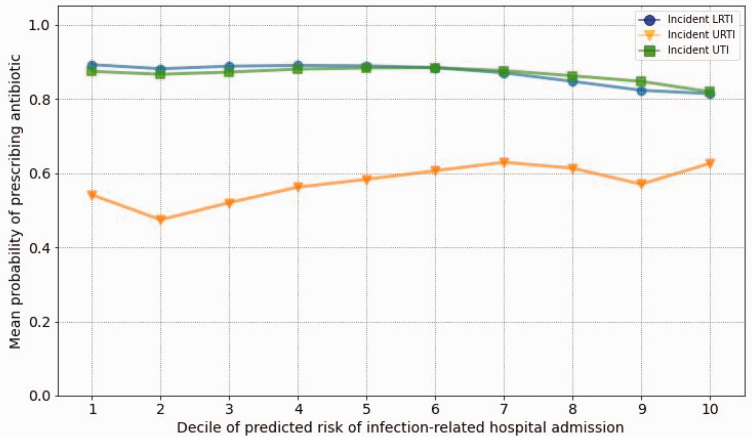
Probability of antibiotic prescribing by predicted risk level of hospital admission related to incident lower respiratory tract infection (LRTI), upper respiratory tract infection (URTI) and urinary tract infection (UTI), where x axis shows deciles of predicted risk of infection-related hospital admissions and y axis shows the mean probability of antibiotic prescribing.

Figure 1 displays the mean probability of antibiotic prescribing by predicted risk of hospital admission in incident LRTI, URTI and UTI (first set of analyses). The probability of being prescribed an antibiotic was unrelated in UTI and LRTI to the risk of infection-related hospital admissions, and weakly related in URTI (from 54% in the lowest to 63% in the highest risk decile). As shown in Table 3, the analyses of AUROCs showed that predicted risk of infection-related hospital admissions was only weakly associated with the probability of antibiotic prescribing (AUROC between 0.54 and 0.56). Supplementary Table S7 shows results of the first analyses for prevalent and other infections. Adjusted ORs also showed no or weak association between risk deciles and probability of being prescribed an antibiotic (Supplementary Tables S8–S10).

**Table 3.** Area under the receiver operating characteristic curve of logistic regression models for antibiotic prescribing with deciles of predicted risk of hospital admissions related to incident and prevalent common infections, including lower respiratory tract infection (LRTI), upper respiratory tract infection (URTI) and urinary tract infection (UTI), using data from January 2019 to March 2022.

	LRTIAUROC	URTIAUROC	UTIAUROC
Development dataset	0.56	0.55	0.54
Validation dataset	0.56	0.55	0.54

AUROC: area under the receiver operating characteristic curve.

Supplementary Table S11 shows the results of the second set of analyses for incident LRTI, URTI and UTI. Patient characteristics such as age and level of co-morbidity were only weakly associated with the probability of being prescribed an antibiotic in incident LRTI, URTI and UTI. The most elderly patients were 31% less likely (adjusted OR: 0.69; 95% CI: 0.69–0.70) to receive an antibiotic for URTI and patients with highest co-morbidity 11% less likely (adjusted OR: 0.89; 95% CI: 0.86–0.91). Probabilities of being prescribed an antibiotic varied between regions. Supplementary Tables S11–S13 show results of the second analyses for prevalent and other infections.

**Figure 2.**
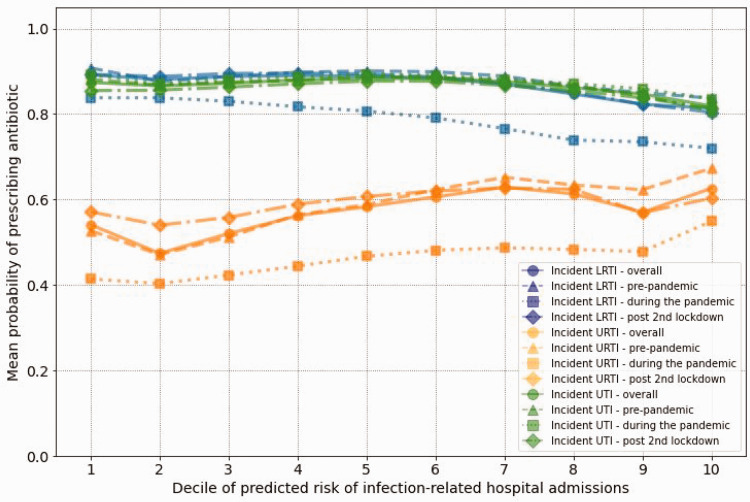
Probability of antibiotic prescribing by predicted risk level of hospital admission related to incident lower respiratory tract infection (LRTI), upper respiratory tract infection (URTI) and urinary tract infection (UTI), stratified by status of COVID-19 pandemic. X axis shows deciles of predicted risk of infection-related hospital admissions and y axis shows the mean probability of antibiotic prescribing.

Figure 2 displays the probability of antibiotic prescribing by predicted risk of hospital admission for incident LRTI, URTI and UTI, stratified by the status of the COVID-19 pandemic. The probability of being prescribed an antibiotic for LRTI and URTI was at the lowest during the pandemic (e.g. 41% in URTI in lowest risk decile). The probability of being prescribed an antibiotic for LRTI and URTI, however, increased after the second national lockdown (e.g. 57% in URTI in lowest risk decile). The probability of being prescribed an antibiotic for UTI faced only minor changes before, during and after the pandemic. Supplementary Figures S2–S4 display the results for probability of antibiotic prescribing for prevalent and other infections. Supplementary Table S14 shows TRIPOD checklist.

## Discussion

### Principal findings

We found that antibiotic prescribing in English primary care was unrelated to prognosis and risk of infection-related hospital admission for LRTI and UTI, and only weakly risk-based for URTI. Patients with the highest age category and highest level of co-morbidity were less likely to be prescribed antibiotics, despite being at higher risk of hospital admission. The COVID-19 pandemic was found to have an impact on both LRTI and URTI, reducing the probability of antibiotic prescribing, given the risk of infection-related hospital admission. A recent study found that the COVID-19 pandemic impacted primary care treatment pathway for common infections – that is, the rate of GP consultations for common infections reduced by 39% in 2020 compared with 2019 and the count of same-day antibiotic prescribing for LRTI reduced by 20%, while that for URTI increased by 15.9% during the first national lockdown.^12^

### Strengths and weaknesses

The main strength of this study was that it used large generalisable EHR data with multiple risk prediction models developed. Another strength of this study was that it evaluated the impact of the COVID-19 pandemic on risk-based prescribing of antibiotics for treatment of common infections. This study had several limitations. One was the absence of consultation type (i.e. in-person or virtual) for infection diagnosis. The latter was common during the COVID-19 pandemic and could have potentially impacted the ability of GPs to assess prognosis. Another limitation was the uncertainty of inclusion into the SGSS of COVID-19 tests performed by private companies. Computational limits constrained dealing with missing values through imputation algorithms, instead of adopting the missing indicator approach. A further limitation was that the risk prediction models did not cover the clinical severity of the infection.

### Comparison with other studies

The latest UK’s AMR strategy includes a target to reduce antibiotic prescribing by 15% by 2024.^13^ A potential challenge is that no cost–benefit assessment has been conducted for the antibiotic reduction targets in England. Lowering the consumption of antibiotics in humans could indeed reduce AMR incidence in the long term but could also lead to increases in infection-related complications. Gulliford et al. showed that general practices can expect an increase in the incidence of pneumonia and peritonsillar abscess if they were to practise policy to reduce antibiotic prescribing for respiratory tract infections.^14^ They advised to exercise caution among subgroups at greater risk of pneumonia. Price et al. supported this, demonstrating an association between recent reductions in antibiotics prescribing for LRTI in primary care and a rise in pneumonia mortality in England and Wales.^15^ Another large data linkage study of 96 health authorities in England also showed significantly fewer hospital admissions and major complications with higher levels of antibiotic prescribing.^16^ This is further reinforced by recent research that found that an increase of 10.4% in general practice antibiotic prescribing (the interquartile range) was associated with a 6% reduction in infection-related hospital admissions.^17^ On the other hand, a time-series analysis concluded that the recent reductions in antibiotic use in primary care was not associated with adverse consequences in the rates of hospital admissions for infections.^18^ However, it seems likely that the rates of complications of a disease would increase if less treatment was administered (unless the treatment is ineffective). Thus, rather than overall targets, there is a need to focus on optimising antibiotic prescribing (i.e. to the right patient at the right time).

Several treatment guidelines for common infections use symptom scores (i.e. sum of weights for different symptoms). Overall, these scores were developed to predict the presence of a bacterial infection, but several studies have shown that some of these symptom scores may only be weak predictors of major clinical outcomes. In a prospective cohort study, it was demonstrated that symptom scores such as CENTOR criteria and FeverPAIN carry low predictive values in identifying patients with acute sore throat who are prone to complications.^19^ Another large cohort study also found that emergency department doctors underestimated the probability of serious bacterial infections (UTI, pneumonia or bacteraemia) in young children with fever.^20^ The present study found that antibiotic prescribing for sore throat was unrelated to risk of infection-related complications, supporting the limited predictive value of symptom scores. In addition to the symptoms, testing C-reactive protein for respiratory tract infections at the point-of-care is believed to reduce unnecessary antibiotic prescribing.^21^ A fundamental question, not addressed in treatment guidelines, is whether the decision to prescribe antibiotics for typically self-limiting infections should be based on prognosis or be symptomatic related to broad set of non-specific symptoms. For sore throat, a Cochrane review reported that the antibiotic effect on symptoms can be small.^22^

A recent study aimed to assess the appropriateness of antibiotic prescribing in primary care and found that about 20% of antibiotic prescribing was inappropriate.^23^ This number informed the target for reduction of antibiotic prescribing in the UK government’s first five-year AMR strategy, to reduce it nationally by 10% by 2020, as part of a greater national target of reducing inappropriate prescribing by 50%.^24^ A limitation of this study was that, apart from the guideline recommendations, no objective clinical definition of appropriateness was provided nor clinical case scenarios for which antibiotics would be inappropriate (in the context of a primary care setting). Furthermore, the treatment guidelines for common infections do not address the frequent challenge of patients returning shortly after an antibiotic was prescribed (except for UTI guideline). Research has found that 29% of patients receive another antibiotic within 30 days.^25^ Rather than targets for reducing inappropriate prescribing, which may be challenging to define, an alternative approach is to focus on improving risk-based antibiotic prescribing for infections that are less severe and typically self-limiting.

### Implications

Antibiotics are routinely indicated for bacterial infections that are unlikely to resolve spontaneously or that have high risks of serious illness and/or complications (e.g. sepsis, pneumonia, peritonsillar abscess or recurrence). For conditions that typically are self-limiting and give on average low risk of complications (e.g. URTI), we feel that several additional criteria should be used to decide on antibiotic treatment. The first one is a higher risk of infection-related complications for a particular patient, given their age, co-morbidity and infection severity. The other one should be antibiotic prescribing history and associated risk of patients harbouring resistant bacteria. Thus, a decision to prescribe an antibiotic is unlikely to be based on one risk threshold (as is done for statins) but weighing of risks and benefits. Rather than a clinical decision-support system that would estimate a general risk score, a clinician could be provided with patient-personalised information about risk of common complications and treatment options according to antibiotic exposure history. Relevant sections of treatment guidelines could also be provided, although most UK treatment guidelines are currently not computable (i.e. cannot be programmed into the EHR).^26^ Also, a leaflet of patient-personalised information could be provided to discuss treatment options with patients and to help dissuade them from using an antibiotic when not needed. Similar leaflets were designed and given to carers for acute cough and respiratory tract infection of children.^27^ Currently, a Knowledge-Support System is providing clinicians and patients with better information (e.g., personalised risk predictions) during each consultation and the effectiveness of this approach is being tested in a cluster trial in north-west England.^28^

## Conclusions

Under the threat of antibiotic overuse and the downstream impacts on developing AMR, many policies have been implemented to reduce overall antibiotic prescribing in primary care based on guidelines and incentivised activities. However, this has not resulted in a greater likelihood of antibiotics being prescribed to patients with higher risks of infection-related hospital admissions, despite substantial differences in risks between those in lowest and highest risk deciles. This was exacerbated during the COVID-19 pandemic since antibiotic prescribing for common infections became even less risk-based. Given the possibility of AMR developing in patients and side-effects with antibiotics, there is a need to better target antibiotics in primary care to patients with worse prognosis. In addition, to explicitly consider prognosis and harm in the treatment guidelines, better personalised information should be provided to clinicians and patients to support shared decision-making with patients.

## Supplemental Material

sj-pdf-1-jrs-10.1177_01410768251328997 - Supplemental material for Antibiotics for common infections in primary care before, during and after the COVID-19 pandemic: cohort study of extent of prescribing based on risks of infection-related hospital admissions Supplemental material, sj-pdf-1-jrs-10.1177_01410768251328997 for Antibiotics for common infections in primary care before, during and after the COVID-19 pandemic: cohort study of extent of prescribing based on risks of infection-related hospital admissions by Ali Fahmi, Ya-Ting Yang, Xiaomin Zhong, Alexander Pate, Anita Sharma, Simon Watts, Darren M Ashcroft, Ben Goldacre, Brian MacKenna, Jon Massey, Amir Mehrkar, Seb Bacon, Brian McMillan, Paul Dark, Kieran Hand, Victoria Palin and Tjeerd Pieter van Staa in Journal of the Royal Society of Medicine

## Declarations

### Guarantor

AF.

### Data availability

NHS England is the data controller of the NHS England OpenSAFELY COVID-19 Service; TPP is the data processor; all study authors using OpenSAFELY have the approval of NHS England (https://digital.nhs.uk/coronavirus/coronavirus-covid-19-response-information-governance-hub/the-nhs-england-opensafely-covid-19-service-privacy-notice). This implementation of OpenSAFELY is hosted within the TPP environment, which is accredited to the ISO 27001 information security standard and is NHS IG Toolkit compliant (https://digital.nhs.uk/data-and-information/looking-after-information/data-security-and-information-governance/data-security-and-protection-toolkit).

Patient data have been pseudonymised for analysis and linkage using industry standard cryptographic hashing techniques; all pseudonymised datasets transmitted for linkage onto OpenSAFELY are encrypted; access to the NHS England OpenSAFELY COVID-19 service is via a virtual private network connection; the researchers hold contracts with NHS England and only access the platform to initiate database queries and statistical models; all database activity is logged; only aggregate statistical outputs leave the platform environment following best practice for anonymisation of results such as statistical disclosure control for low cell counts (https://digital.nhs.uk/data-and-information/information-standards/information-standards-and-data-collections-including-extractions/publications-and-notifications/standards-and-collections/isb1523-anonymisation-standard-for-publishing-health-and-social-care-data).

The service adheres to the obligations of the UK General Data Protection Regulation (UK GDPR) and the Data Protection Act 2018. The service previously operated under notices initially issued in February 2020 by the Secretary of State under Regulation 3(4) of the Health Service (Control of Patient Information) Regulations 2002 (COPI Regulations), which required organisations to process confidential patient information for COVID-19 purposes; this set aside the requirement for patient consent (https://www.gov.uk/government/publications/coronavirus-covid-19-notification-of-data-controllers-to-share-information/5de48745-c52e-4799-898f-f617c43c3930). As of 1 July 2023, the Secretary of State has requested that NHS England continue to operate the Service under the COVID-19 Directions 2020 (https://digital.nhs.uk/about-nhs-digital/corporate-information-and-documents/directions-and-data-provision-notices/secretary-of-state-directions/covid-19-public-health-directions-2020). In some cases of data sharing, the common law duty of confidence is met using, for example, patient consent or support from the Health Research Authority Confidentiality Advisory Group (https://www.hra.nhs.uk/about-us/committees-and-services/confidentiality-advisory-group/).

Taken together, these provide the legal bases to link patient datasets using the service. GP practices, which provide access to the primary care data, are required to share relevant health information to support the public health response to the pandemic, and have been informed of how the service operates.

Access to the underlying identifiable and potentially re-identifiable pseudonymised EHR data is tightly governed by various legislative and regulatory frameworks and restricted by best practice. The data in OpenSAFELY are drawn from General Practice data across England where TPP is the Data Processor. TPP developers initiate an automated process to create pseudonymised records in the core OpenSAFELY database, which are copies of key structured data tables in the identifiable records. These are linked onto key external data resources that have also been pseudonymised via SHA-512 one-way hashing of NHS numbers using a shared salt. DataLab developers and PIs holding contracts with NHS England have access to the OpenSAFELY pseudonymised data tables as needed to develop the OpenSAFELY tools. These tools in turn enable researchers with OpenSAFELY Data Access Agreements to write and execute code for data management and data analysis without direct access to the underlying raw pseudonymised patient data, and to review the outputs of this code. All code for the full data management pipeline – from raw data to completed results for this analysis – and for the OpenSAFELY platform as a whole is available for review at github.com/OpenSAFELY.

All data were linked, stored and analysed securely within the OpenSAFELY platform https://opensafely.org/. Data include pseudonymised data such as coded diagnoses, medications and physiological parameters. No free text data are included. All code is shared openly for review and re-use under MIT open license (https://github.com/opensafely/amr-uom-brit/). Detailed pseudonymised patient data are potentially re-identifiable and therefore not shared. We rapidly delivered the OpenSAFELY data analysis platform without prior funding to deliver timely analyses on urgent research questions in the context of the global COVID-19 health emergency: now that the platform is established, we are developing a formal process for external users to request access in collaboration with NHS England; details of this process will be published shortly on OpenSAFELY.org.

### Provenance

Commissioned; peer-reviewed by Julie Morris and Elizabeth Haworth.
