# Supplementary material for: Antibiotics for common infections in primary care before, during and after the COVID-19 pandemic: cohort study of extent of prescribing based on risks of infection-related hospital admissions
Source: J R Soc Med. 2025 Apr 3;118(4):126–37. doi: 10.1177/01410768251328997 (PMC11969485; doi:10.1177/01410768251328997)
Supplement: sj-pdf-1-jrs-10.1177_01410768251328997 - Supplemental material for Antibiotics for common infections in primary care before, during and after the COVID-19 pandemic: cohort study of extent of prescribing based on risks of infection-related hospital admissions [file sj-pdf-1-jrs-10.1177_01410768251328997.pdf]

Supplementary materials

Table of Contents

Supplementary Tables 2

Baseline characteristics 2

Counts and rates of hospital admission cases 9

Logistic regression models 12

Performance 12

Odds ratios 13

Supplementary Figures 21

TRIPOD checklist 23

Supplementary Tables

Baseline characteristics

Table S1. Further baseline characteristics of cohort of common infections, including lower respiratory tract infection (LRTI), upper respiratory tract infection (URTI), and urinary tract infection (UTI), using data from January 2019 to March 2023. The cohort consists of incident infections with no prescribed antibiotics, incident infections with prescribed antibiotics, prevalent infections with no prescribed antibiotics, and prevalent infections with prescribed antibiotics.

	LRTI				URTI				UTI			
	Incident		Prevalent		Incident		Prevalent		Incident		Prevalent	
	No ABs[1]	With ABs	No ABs	With ABs	No ABs	With ABs	No ABs	With ABs	No ABs	With ABs	No ABs	With ABs
BMI[2], N (%)												
Underweight	8,585 (2.69)	35,930 (1.72)	1,915 (2.59)	4,045 (1.99)	65,760 (2.15)	71,175 (1.70)	6,305 (1.85)	7,050 (1.91)	7,640 (2.18)	42,035 (1.85)	1,855 (2.02)	5,690 (1.82)
Healthy weight	77,385 (24.28)	431,010 (20.63)	17,930 (24.31)	42,705 (21.05)	738,360 (24.10)	878,775 (21.04)	77,170 (22.64)	78,380 (21.22)	92,630 (26.38)	598,440 (26.32)	24,360 (26.53)	80,705 (25.80)
Overweight	83,165 (26.09)	558,020 (26.70)	19,705 (26.71)	55,265 (27.24)	847,185 (27.66)	1,077,800 (25.80)	93,925 (27.56)	99,095 (26.83)	90,915 (25.89)	598,375 (26.32)	25,240 (27.49)	86,620 (27.69)
Obese	88,945 (27.90)	659,385 (31.56)	21,740 (29.48)	68,545 (33.78)	866,815 (28.30)	1,267,850 (30.35)	102,265 (30.00)	121,260 (32.83)	88,490 (25.20)	589,340 (25.92)	23,775 (25.90)	84,630 (27.06)
Unknown	60,695 (19.04)	405,260 (19.39)	12,470 (16.91)	32,355 (15.95)	545,230 (17.80)	881,770 (21.11)	61,170 (17.95)	63,545 (17.21)	71,475 (20.35)	445,635 (19.60)	16,580 (18.06)	55,165 (17.64)
Smoking status, N (%)												
Smoker	51,915 (16.29)	395,535 (18.93)	10,300 (13.97)	34,615 (17.06)	648,830 (21.18)	796,990 (19.08)	56,340 (16.53)	66,625 (18.04)	40,990 (11.67)	257,225 (11.31)	8,585 (9.35)	29,470 (9.42)
Ex-smoker	160,505 (50.35)	983,925 (47.09)	38,820 (52.63)	105,400 (51.94)	1,466,735 (47.88)	1,861,565 (44.56)	164,805 (48.35)	180,870 (48.97)	162,260 (46.21)	1,036,665 (45.59)	45,035 (49.05)	153,790 (49.16)
Never smoked	105,160 (32.99)	703,900 (33.69)	24,490 (33.20)	62,645 (30.87)	933,050 (30.46)	1,494,055 (35.77)	118,535 (34.78)	120,650 (32.67)	146,285 (41.66)	973,185 (42.80)	37,925 (41.31)	128,940 (41.22)
Unknown	1,190 (0.37)	6,240 (0.30)	145 (0.20)	260 (0.13)	14,735 (0.48)	24,760 (0.59)	1,160 (0.34)	1,170 (0.32)	1,615 (0.46)	6,745 (0.30)	260 (0.28)	605 (0.19)
IMD[3], N (%)												
1 (most deprived)	52,475 (16.46)	326,660 (15.63)	13,400 (18.17)	31,870 (15.71)	475,755 (15.53)	642,865 (15.39)	59,090 (17.34)	57,615 (15.60)	64,150 (18.27)	442,440 (19.46)	19,205 (20.92)	65,470 (20.93)
2	58,600 (18.38)	381,805 (18.27)	14,315 (19.41)	37,605 (18.53)	546,470 (17.84)	746,935 (17.88)	63,840 (18.73)	66,470 (18.00)	68,765 (19.58)	462,900 (20.36)	19,255 (20.97)	66,015 (21.10)
3	65,170 (20.44)	417,445 (19.98)	15,315 (20.76)	40,520 (19.97)	624,610 (20.39)	837,245 (20.04)	71,550 (20.99)	75,635 (20.48)	73,165 (20.84)	474,485 (20.87)	19,585 (21.33)	65,740 (21.02)
4	62,795 (19.70)	421,775 (20.18)	14,020 (19.01)	40,425 (19.92)	615,540 (20.09)	869,130 (20.81)	65,450 (19.20)	74,885 (20.28)	67,970 (19.36)	424,170 (18.65)	16,430 (17.90)	56,005 (17.90)
5 (most affluent)	74,615 (23.41)	506,725 (24.25)	15,535 (21.06)	48,945 (24.12)	750,935 (24.51)	1,013,810 (24.27)	75,370 (22.11)	88,420 (23.94)	71,315 (20.31)	434,365 (19.10)	15,865 (17.28)	54,610 (17.46)

Unknown	5,125 (1.61)	35,195 (1.68)	1,175 (1.59)	3,550 (1.75)	50,030 (1.63)	67,380 (1.61)	5,545 (1.63)	6,295 (1.70)	5,785 (1.65)	35,460 (1.56)	1,465 (1.60)	4,960 (1.59)
Season, N (%)												
Spring	77,760 (24.39)	467,770 (22.39)	17,910 (24.28)	46,605 (22.97)	757,260 (24.72)	978,295 (23.42)	90,955 (26.69)	91,420 (24.75)	88,190 (25.12)	570,710 (25.10)	23,105 (25.16)	79,140 (25.30)
Summer	53,645 (16.83)	281,925 (13.49)	11,165 (15.14)	26,970 (13.29)	580,820 (18.96)	626,605 (15.00)	61,585 (18.07)	53,530 (14.49)	83,060 (23.65)	538,895 (23.70)	21,765 (23.71)	73,075 (23.36)
Autumn	73,360 (23.01)	496,470 (23.76)	15,635 (21.20)	43,785 (21.58)	726,980 (23.73)	992,730 (23.76)	74,575 (21.88)	80,465 (21.79)	86,115 (24.52)	557,260 (24.51)	22,720 (24.75)	76,395 (24.42)
Winter	114,010 (35.76)	843,440 (40.36)	29,045 (39.38)	85,555 (42.16)	998,285 (32.59)	1,579,760 (37.82)	113,715 (33.36)	143,905 (38.96)	93,785 (26.71)	606,955 (26.69)	24,220 (26.38)	84,195 (26.92)
Region, N (%)												
London	11,960 (3.75)	63,035 (3.02)	1,930 (2.62)	3,560 (1.75)	117,625 (3.84)	184,635 (4.42)	14,805 (4.34)	12,250 (3.32)	14,730 (4.19)	69,720 (3.07)	3,145 (3.42)	6,865 (2.19)
North East	19,900 (6.24)	111,235 (5.32)	4,340 (5.89)	10,740 (5.29)	198,130 (6.47)	213,180 (5.10)	19,935 (5.85)	19,570 (5.30)	19,080 (5.43)	99,280 (4.37)	4,305 (4.69)	13,260 (4.24)
North West	36,190 (11.35)	232,825 (11.14)	9,205 (12.48)	25,880 (12.75)	371,295 (12.12)	445,700 (10.67)	42,010 (12.32)	45,525 (12.33)	38,130 (10.86)	244,800 (10.77)	10,355 (11.28)	38,035 (12.16)
East	72,835 (22.85)	482,505 (23.09)	15,865 (21.51)	44,835 (22.09)	660,655 (21.57)	1,014,605 (24.29)	71,545 (20.99)	84,665 (22.92)	81,600 (23.24)	538,410 (23.68)	19,960 (21.74)	71,530 (22.87)
West Midlands	14,455 (4.53)	100,140 (4.79)	3,060 (4.15)	8,740 (4.31)	120,275 (3.93)	213,115 (5.10)	14,005 (4.11)	16,690 (4.52)	14,270 (4.06)	96,675 (4.25)	3,340 (3.64)	12,650 (4.04)
Yorkshire and The Humber	51,090 (16.03)	398,720 (19.08)	14,080 (19.09)	40,750 (20.08)	496,960 (16.22)	719,445 (17.22)	59,750 (17.53)	64,635 (17.50)	51,975 (14.80)	368,810 (16.22)	17,045 (18.56)	53,565 (17.12)
South East	19,840 (6.22)	110,815 (5.30)	3,965 (5.38)	9,945 (4.90)	187,755 (6.13)	232,750 (5.57)	20,380 (5.98)	21,260 (5.76)	23,835 (6.79)	138,110 (6.07)	5,500 (5.99)	18,090 (5.78)
East Midlands	55,020 (17.26)	397,695 (19.03)	13,770 (18.67)	42,760 (21.07)	541,370 (17.67)	760,170 (18.20)	62,325 (18.29)	71,795 (19.44)	60,180 (17.14)	449,370 (19.76)	17,015 (18.53)	66,385 (21.22)
South West	37,490 (11.76)	192,630 (9.22)	7,540 (10.22)	15,710 (7.74)	369,285 (12.05)	393,775 (9.43)	36,080 (10.59)	32,940 (8.92)	47,345 (13.48)	268,650 (11.81)	11,155 (12.15)	32,420 (10.36)

[1] ABs, antibiotics prescribed or not.
[2] BMI, Body Mass Index recorded in the last 5 years.
[3] IMD, Multiple Deprivation Index, quintile measured from patient-level address.

Table S2. Baseline characteristics of cohort of other common infections, including sinusitis, otitis media, and otitis externa, using data from January 2019 to March 2023. The cohort consists of incident infections with no prescribed antibiotics, incident infections with prescribed antibiotics, prevalent infections with no prescribed antibiotics, and prevalent infections with prescribed antibiotics.

	Sinusitis				Otitis media				Otitis externa			
	Incident		Prevalent		Incident		Incident		Prevalent		Incident	
	No ABs[1]	With ABs	No ABs	With ABs	No ABs	With ABs	No ABs	With ABs	No ABs	With ABs	No ABs	With ABs
**Total, N cases**	158,780	457,790	17,350	32,550	697,730	187,555	68,015	27,470	97,105	278,855	12,040	18,995
**Age, N (%)**												
18-24	11,770 (7.41)	25,365 (5.54)	970 (5.58)	1,395 (4.28)	58,085 (8.33)	16,025 (8.54)	5,245 (7.71)	2,005 (7.29)	10,190 (10.49)	29,740 (10.67)	1,075 (8.92)	1,660 (8.75)
25-34	29,910 (18.84)	78,790 (17.21)	2,880 (16.59)	4,955 (15.22)	110,610 (15.85)	33,160 (17.68)	9,985 (14.68)	4,525 (16.47)	18,245 (18.79)	58,510 (20.98)	2,230 (18.54)	3,940 (20.73)
35-44	30,765 (19.38)	92,025 (20.10)	3,435 (19.79)	6,685 (20.54)	108,920 (15.61)	32,760 (17.47)	9,805 (14.42)	4,480 (16.31)	15,895 (16.37)	52,610 (18.87)	2,055 (17.07)	3,595 (18.94)
45-54	31,420 (19.79)	97,965 (21.40)	3,775 (21.77)	7,435 (22.83)	126,905 (18.19)	36,440 (19.43)	11,865 (17.44)	5,015 (18.26)	16,700 (17.20)	50,940 (18.27)	2,230 (18.52)	3,600 (18.96)
55-64	26,850 (16.91)	84,295 (18.41)	3,140 (18.10)	6,230 (19.15)	117,815 (16.89)	30,895 (16.47)	11,340 (16.67)	4,590 (16.71)	14,960 (15.41)	40,905 (14.67)	1,885 (15.67)	2,885 (15.18)
65-74	19,585 (12.34)	57,540 (12.57)	2,285 (13.18)	4,355 (13.39)	104,220 (14.94)	23,535 (12.55)	10,895 (16.02)	3,890 (14.15)	12,395 (12.76)	29,275 (10.50)	1,485 (12.35)	2,105 (11.07)
75+	8,485 (5.34)	21,810 (4.76)	865 (5.00)	1,495 (4.59)	71,170 (10.20)	14,745 (7.86)	8,875 (13.05)	2,965 (10.80)	8,720 (8.98)	16,865 (6.05)	1,075 (8.92)	1,210 (6.37)
**Sex, N (%)**												
Female	103,475 (65.17)	337,195 (73.66)	11,925 (68.74)	24,200 (74.35)	409,790 (58.73)	118,370 (63.11)	39,410 (57.94)	16,675 (60.70)	57,560 (59.28)	181,785 (65.19)	7,320 (60.80)	12,125 (63.83)
Male	55,305 (34.83)	120,595 (26.34)	5,425 (31.26)	8,350 (25.65)	287,940 (41.27)	69,185 (36.89)	28,605 (42.06)	10,795 (39.30)	39,545 (40.72)	97,070 (34.81)	4,720 (39.20)	6,870 (36.17)
**BMI[2], N (%)**												
Underweight	2,140 (1.35)	5,925 (1.29)	225 (1.30)	455 (1.39)	8,415 (1.21)	2,125 (1.13)	800 (1.17)	300 (1.08)	1,250 (1.29)	3,465 (1.24)	135 (1.12)	220 (1.16)
Healthy weight	39,350 (24.78)	109,060 (23.82)	4,315 (24.86)	7,715 (23.70)	144,985 (20.78)	35,315 (18.83)	13,875 (20.40)	5,230 (19.04)	20,695 (21.31)	54,715 (19.62)	2,455 (20.41)	3,495 (18.40)
Overweight	41,380 (26.06)	116,625 (25.48)	4,600 (26.50)	8,465 (26.01)	179,925 (25.79)	45,545 (24.28)	18,125 (26.65)	7,005 (25.51)	23,820 (24.53)	66,770 (23.95)	2,955 (24.56)	4,650 (24.47)
Obese	37,510 (23.62)	119,235 (26.05)	4,320 (24.89)	9,000 (27.65)	189,875 (27.21)	57,855 (30.85)	19,595 (28.81)	8,620 (31.38)	25,610 (26.37)	80,330 (28.81)	3,420 (28.39)	5,815 (30.61)
Unknown	38,395 (24.18)	106,945 (23.36)	3,895 (22.45)	6,920 (21.25)	174,525 (25.01)	46,720 (24.91)	15,615 (22.96)	6,320 (23.00)	25,725 (26.49)	73,575 (26.38)	3,070 (25.52)	4,815 (25.36)
**Ethnicity, N (%)**												
White	120,790 (76.07)	354,610 (77.46)	13,755 (79.29)	26,625 (81.80)	522,990 (74.96)	144,755 (77.18)	53,985 (79.37)	22,025 (80.16)	73,880 (76.08)	208,435 (74.75)	9,320 (77.42)	14,805 (77.95)
Asian	8,125 (5.12)	20,525 (4.48)	760 (4.37)	1,175 (3.61)	37,415 (5.36)	9,845 (5.25)	3,110 (4.57)	1,270 (4.62)	6,050 (6.23)	18,600 (6.67)	785 (6.52)	1,230 (6.48)
Black	2,000 (1.26)	4,210 (0.92)	175 (1.00)	225 (0.69)	7,640 (1.09)	1,720 (0.92)	635 (0.94)	215 (0.79)	1,250 (1.29)	3,030 (1.09)	110 (0.91)	170 (0.88)
Mixed	1,565 (0.99)	3,985 (0.87)	160 (0.92)	285 (0.87)	5,970 (0.86)	1,550 (0.83)	510 (0.75)	195 (0.71)	905 (0.93)	2,500 (0.90)	85 (0.70)	175 (0.92)
Other	2,785 (1.75)	6,045 (1.32)	285 (1.63)	385 (1.18)	10,965 (1.57)	2,590 (1.38)	1,000 (1.47)	305 (1.12)	1,595 (1.64)	4,310 (1.55)	175 (1.45)	290 (1.52)
Unknown	23,520 (14.81)	68,415 (14.94)	2,220 (12.79)	3,860 (11.85)	112,750 (16.16)	27,095 (14.45)	8,780 (12.91)	3,465 (12.61)	13,420 (13.82)	41,980 (15.05)	1,565 (13.00)	2,325 (12.25)
**CCI[3], N (%)**												
Very low (=0)	115,320 (72.63)	328,330 (71.72)	12,295 (70.85)	22,245 (68.34)	503,130 (72.11)	132,910 (70.86)	46,975 (69.07)	18,735 (68.20)	70,565 (72.67)	204,305 (73.27)	8,620 (71.58)	13,620 (71.72)
Low (=1 and =2)	37,855 (23.84)	112,130 (24.49)	4,350 (25.08)	8,935 (27.45)	159,260 (22.83)	44,920 (23.95)	16,520 (24.29)	6,900 (25.11)	21,905 (22.56)	62,955 (22.58)	2,800 (23.27)	4,520 (23.80)
Medium (=3 and =4)	4,640 (2.92)	14,530 (3.17)	595 (3.44)	1,145 (3.52)	28,845 (4.13)	7,930 (4.23)	3,585 (5.27)	1,485 (5.40)	3,740 (3.85)	9,520 (3.41)	500 (4.17)	675 (3.55)
High (=5 and =6)	760 (0.48)	2,160 (0.47)	90 (0.51)	175 (0.53)	5,095 (0.73)	1,410 (0.75)	755 (1.11)	285 (1.03)	680 (0.70)	1,625 (0.58)	90 (0.75)	130 (0.68)
Very high (≥7)	205 (0.13)	640 (0.14)	20 (0.12)	55 (0.16)	1,400 (0.20)	390 (0.21)	185 (0.27)	70 (0.26)	215 (0.22)	445 (0.16)	25 (0.22)	45 (0.25)
**Smoking status, N (%)**												
Smoker	22,645 (14.26)	70,885 (15.48)	2,450 (14.13)	5,115 (15.72)	111,280 (15.95)	37,295 (19.89)	10,525 (15.47)	4,790 (17.43)	16,980 (17.49)	53,980 (19.36)	2,105 (17.48)	3,515 (18.52)
Ex-smoker	67,025 (42.21)	194,600 (42.51)	7,570 (43.62)	14,320 (44.00)	295,765 (42.39)	78,175 (41.68)	29,735 (43.72)	12,075 (43.94)	39,075 (40.24)	109,140 (39.14)	4,890 (40.61)	7,790 (41.02)
Never smoked	68,195 (42.95)	190,370 (41.58)	7,270 (41.91)	13,035 (40.04)	285,065 (40.86)	70,735 (37.71)	27,330 (40.19)	10,460 (38.08)	40,095 (41.29)	113,205 (40.60)	4,970 (41.28)	7,550 (39.76)
Unknown	915 (0.58)	1,930 (0.42)	60 (0.35)	80 (0.24)	5,615 (0.80)	1,350 (0.72)	420 (0.62)	150 (0.55)	955 (0.98)	2,530 (0.91)	75 (0.63)	135 (0.71)
**IMD[4], N (%)**												
1 (most deprived)	30,200 (19.02)	88,755 (19.39)	3,475 (20.03)	6,375 (19.59)	129,290 (18.53)	30,595 (16.31)	13,565 (19.95)	5,005 (18.22)	16,045 (16.53)	42,190 (15.13)	2,205 (18.31)	2,920 (15.38)
2	32,265 (20.32)	95,665 (20.90)	3,515 (20.26)	6,910 (21.23)	136,250 (19.53)	34,710 (18.51)	13,855 (20.37)	5,515 (20.07)	17,830 (18.36)	49,465 (17.74)	2,265 (18.82)	3,495 (18.40)
3	33,100 (20.85)	97,095 (21.21)	3,715 (21.42)	6,875 (21.12)	142,980 (20.49)	38,125 (20.33)	13,985 (20.57)	5,695 (20.72)	19,335 (19.91)	54,565 (19.57)	2,450 (20.35)	3,610 (19.01)
4	30,025 (18.91)	86,900 (18.98)	3,230 (18.62)	6,100 (18.75)	133,005 (19.06)	37,985 (20.25)	12,225 (17.97)	5,145 (18.73)	19,635 (20.22)	58,175 (20.86)	2,250 (18.68)	3,920 (20.64)
5 (most affluent)	30,510 (19.22)	81,930 (17.90)	3,120 (17.99)	5,740 (17.63)	145,210 (20.81)	43,110 (22.99)	13,280 (19.53)	5,675 (20.65)	22,640 (23.31)	69,885 (25.06)	2,675 (22.21)	4,735 (24.94)
Unknown	2,680 (1.69)	7,450 (1.63)	295 (1.69)	550 (1.69)	10,995 (1.58)	3,030 (1.61)	1,100 (1.62)	445 (1.62)	1,620 (1.67)	4,575 (1.64)	195 (1.63)	310 (1.62)
**Season, N (%)**												
Spring	41,800 (26.33)	118,335 (25.85)	4,935 (28.43)	8,915 (27.39)	162,350 (23.27)	43,525 (23.21)	15,185 (22.32)	5,995 (21.82)	23,165 (23.86)	66,780 (23.95)	2,845 (23.63)	4,575 (24.10)

Summer	26,625 (16.77)	67,750 (14.80)	2,905 (16.73)	4,810 (14.78)	165,510 (23.72)	43,455 (23.17)	16,220 (23.85)	6,605 (24.05)	21,180 (21.81)	56,750 (20.35)	2,440 (20.28)	3,895 (20.50)
Autumn	36,755 (23.15)	102,395 (22.37)	3,820 (22.01)	7,025 (21.58)	175,270 (25.12)	46,990 (25.05)	18,105 (26.62)	7,285 (26.52)	23,050 (23.74)	65,630 (23.54)	2,835 (23.55)	4,405 (23.20)
Winter	53,595 (33.76)	169,310 (36.98)	5,695 (32.83)	11,800 (36.25)	194,595 (27.89)	53,585 (28.57)	18,500 (27.20)	7,585 (27.61)	29,710 (30.59)	89,690 (32.16)	3,915 (32.54)	6,115 (32.21)
**Region, N (%)**												
London	7,190 (4.53)	15,995 (3.49)	665 (3.82)	820 (2.52)	27,855 (3.99)	7,105 (3.79)	2,460 (3.62)	810 (2.94)	4,305 (4.43)	10,190 (3.65)	465 (3.85)	550 (2.91)
North East	8,540 (5.38)	19,430 (4.24)	890 (5.14)	1,280 (3.94)	36,480 (5.23)	9,500 (5.07)	3,575 (5.26)	1,475 (5.37)	5,660 (5.83)	13,715 (4.92)	605 (5.02)	945 (4.98)
North West	19,650 (12.38)	48,555 (10.61)	2,360 (13.60)	4,040 (12.41)	78,420 (11.24)	19,225 (10.25)	8,115 (11.93)	3,020 (10.99)	11,130 (11.46)	30,340 (10.88)	1,470 (12.21)	2,245 (11.82)
East	35,940 (22.63)	124,515 (27.20)	3,995 (23.02)	8,670 (26.64)	154,940 (22.21)	47,385 (25.26)	14,650 (21.54)	6,565 (23.89)	25,295 (26.05)	73,250 (26.27)	3,120 (25.90)	5,010 (26.37)
West Midlands	5,590 (3.52)	16,220 (3.54)	565 (3.24)	1,000 (3.08)	25,790 (3.70)	7,185 (3.83)	2,050 (3.01)	855 (3.12)	4,295 (4.42)	12,675 (4.55)	520 (4.34)	860 (4.54)
Yorkshire and The Humber	27,295 (17.19)	73,225 (15.99)	3,105 (17.90)	5,480 (16.84)	116,575 (16.71)	30,210 (16.11)	11,775 (17.31)	4,515 (16.43)	16,395 (16.88)	48,930 (17.55)	2,185 (18.13)	3,340 (17.59)
South East	10,280 (6.47)	28,055 (6.13)	950 (5.48)	1,955 (6.00)	45,650 (6.54)	11,465 (6.11)	4,705 (6.92)	1,800 (6.55)	5,280 (5.44)	13,895 (4.98)	610 (5.07)	900 (4.74)
East Midlands	26,150 (16.47)	84,450 (18.45)	3,180 (18.34)	6,455 (19.82)	130,300 (18.68)	36,600 (19.52)	12,980 (19.08)	5,700 (20.75)	16,010 (16.49)	52,870 (18.96)	2,190 (18.20)	3,905 (20.55)
South West	18,150 (11.43)	47,345 (10.34)	1,640 (9.46)	2,850 (8.76)	81,715 (11.71)	18,880 (10.07)	7,700 (11.32)	2,735 (9.95)	8,740 (9.00)	22,990 (8.25)	880 (7.29)	1,235 (6.51)
**Flu vaccination, N (%)**												
Yes	44,700 (28.15)	134,720 (29.43)	5,255 (30.29)	10,595 (32.54)	228,750 (32.79)	57,560 (30.69)	25,105 (36.92)	9,700 (35.31)	28,805 (29.66)	75,175 (26.96)	3,695 (30.68)	5,555 (29.25)
No	114,080 (71.85)	323,070 (70.57)	12,095 (69.71)	21,960 (67.46)	468,975 (67.21)	130,000 (69.31)	42,905 (63.08)	17,775 (64.69)	68,300 (70.34)	203,675 (73.04)	8,345 (69.32)	13,440 (70.75)
**Period**												
Pre-pandemic	51,265 (32.29)	143,165 (31.27)	5,360 (30.90)	10,120 (31.09)	225,470 (32.32)	55,475 (29.58)	22,850 (33.60)	9,350 (34.04)	29,900 (30.79)	83,505 (29.95)	3,950 (32.80)	6,170 (32.48)
During pandemic	23,460 (14.78)	59,490 (13.00)	3,145 (18.12)	5,270 (16.19)	130,420 (18.69)	38,375 (20.46)	13,730 (20.18)	5,725 (20.84)	17,435 (17.95)	50,100 (17.97)	2,220 (18.42)	3,455 (18.18)
After 2nd lockdown	67,670 (42.62)	207,150 (45.25)	6,980 (40.23)	13,510 (41.50)	284,410 (40.76)	77,680 (41.42)	25,985 (38.21)	9,970 (36.29)	41,420 (42.65)	119,035 (42.69)	4,650 (38.63)	7,375 (38.84)
**Count of antibiotic prescription, mean (SD[5])**	1.16 (1.70)	2.31 (1.73)	2.41 (1.97)	3.54 (1.93)	0.91 (1.39)	2.05 (1.47)	1.51 (1.58)	2.49 (1.50)	1.08 (1.56)	2.07 (1.49)	2.08 (1.65)	3.07 (1.54)

[1] ABs, antibiotics prescribed or not.
[2] BMI, Body Mass Index recorded in the last 5 years.
[3] CCI, Charlson Comorbidities Index, measured from 17 weighted conditions, including myocardial infarction, congestive heart failure, peripheral vascular disease, cerebrovascular disease, dementia, chronic pulmonary disease, Connective tissue disease, ulcer disease, mild liver disease, diabetes, hemiplegia, moderate or severe renal disease, diabetes with complications, any malignancy (including leukaemia and lymphoma), moderate or severe liver disease, metastatic solid tumour, and AIDS.
[4] IMD, Multiple Deprivation Index, quintile measured from patient-level address.
[5] SD, standard deviation.

Table S3. Baseline characteristics of cohort of URTI infections, including specific upper respiratory tract infection (URTI), cough, cold with cough, and sore throat, using data from January 2019 to March 2023. The cohort consists of incident infections with no prescribed antibiotics, incident infections with prescribed antibiotics, prevalent infections with no prescribed antibiotics, and prevalent infections with prescribed antibiotics.

	Specific URTI				Cough				Cold with cough				Sore throat			
	Incident		Prevalent		Incident		Prevalent		Incident		Prevalent		Incident		Prevalent	
	No ABs[1]	With ABs	No ABs	With ABs	No ABs	With ABs	No ABs	With ABs	No ABs	With ABs	No ABs	With ABs	No ABs	With ABs	No ABs	With ABs
Total, N cases	261,130	535,940	11,810	21,230	1,055,800	755,950	92,900	55,880	1,419,445	2,211,690	199,735	254,600	326,970	673,800	36,385	37,615
Age, N (%)																
18-24	28,195 (10.80)	32,640 (6.09)	785 (6.63)	830 (3.91)	39,630 (3.75)	32,850 (4.35)	2,915 (3.14)	1,560 (2.79)	60,845 (4.29)	90,715 (4.10)	5,995 (3.00)	5,825 (2.29)	62,175 (19.02)	161,985 (24.04)	9,115 (25.05)	11,115 (29.55)
25-34	48,605 (18.61)	71,205 (13.29)	1,610 (13.64)	2,145 (10.10)	75,150 (7.12)	75,445 (9.98)	6,555 (7.05)	4,190 (7.50)	116,005 (8.17)	212,460 (9.61)	14,525 (7.27)	16,815 (6.60)	81,130 (24.81)	209,415 (31.08)	10,125 (27.83)	12,215 (32.47)
35-44	43,905 (16.81)	78,880 (14.72)	1,810 (15.31)	2,740 (12.90)	88,520 (8.38)	88,095 (11.65)	8,785 (9.46)	5,520 (9.88)	130,500 (9.19)	253,730 (11.47)	19,140 (9.58)	23,580 (9.26)	59,085 (18.07)	127,265 (18.89)	5,995 (16.48)	6,515 (17.32)
45-54	44,365 (16.99)	93,255 (17.40)	2,130 (18.02)	3,590 (16.92)	152,650 (14.46)	124,575 (16.48)	15,230 (16.39)	8,885 (15.90)	204,695 (14.42)	357,025 (16.14)	30,775 (15.41)	38,005 (14.93)	47,650 (14.57)	80,760 (11.99)	4,480 (12.31)	3,695 (9.82)
55-64	39,395 (15.09)	97,645 (18.22)	2,135 (18.08)	4,090 (19.26)	223,240 (21.14)	151,775 (20.08)	20,265 (21.81)	11,635 (20.82)	280,450 (19.76)	425,770 (19.25)	39,730 (19.89)	49,995 (19.64)	36,365 (11.12)	50,870 (7.55)	3,100 (8.51)	2,240 (5.96)
65-74	30,205 (11.57)	86,200 (16.08)	1,615 (13.67)	3,950 (18.60)	267,615 (25.35)	152,795 (20.21)	21,630 (23.28)	12,700 (22.73)	330,115 (23.26)	433,290 (19.59)	43,055 (21.56)	56,525 (22.20)	25,200 (7.71)	28,400 (4.22)	2,275 (6.26)	1,220 (3.24)
75+	26,460 (10.13)	76,115 (14.20)	1,730 (14.65)	3,885 (18.30)	208,990 (19.79)	130,415 (17.25)	17,530 (18.87)	11,390 (20.38)	296,830 (20.91)	438,695 (19.84)	46,520 (23.29)	63,845 (25.08)	15,360 (4.70)	15,105 (2.24)	1,295 (3.56)	615 (1.64)
Sex, N (%)																
Female	170,235 (65.19)	339,975 (63.44)	8,090 (68.49)	13,875 (65.36)	571,925 (54.17)	457,310 (60.49)	53,640 (57.74)	34,415 (61.58)	777,180 (54.75)	1,336,250 (60.42)	116,900 (58.53)	155,950 (61.25)	215,915 (66.03)	473,185 (70.23)	24,140 (66.35)	27,270 (72.49)
Male	90,895 (34.81)	195,965 (36.56)	3,720 (31.51)	7,355 (34.64)	483,875 (45.83)	298,640 (39.51)	39,260 (42.26)	21,465 (38.42)	642,265 (45.25)	875,440 (39.58)	82,835 (41.47)	98,650 (38.75)	111,055 (33.97)	200,615 (29.77)	12,245 (33.65)	10,345 (27.51)
BMI[2], N (%)																
Underweight	4,215 (1.61)	8,440 (1.58)	220 (1.86)	325 (1.53)	23,775 (2.25)	13,435 (1.78)	1,560 (1.68)	1,065 (1.91)	32,190 (2.27)	38,865 (1.76)	3,890 (1.95)	5,005 (1.97)	5,580 (1.71)	10,435 (1.55)	635 (1.75)	655 (1.74)
Healthy weight	59,390 (22.74)	110,805 (20.68)	2,545 (21.54)	4,400 (20.72)	256,965 (24.34)	158,570 (20.98)	20,655 (22.23)	11,640 (20.83)	344,120 (24.24)	460,360 (20.81)	45,230 (22.64)	53,880 (21.16)	77,885 (23.82)	149,040 (22.12)	8,740 (24.03)	8,460 (22.49)
Overweight	64,175 (24.58)	140,120 (26.14)	3,070 (26.00)	5,685 (26.77)	306,630 (29.04)	203,430 (26.91)	26,835 (28.88)	15,505 (27.75)	402,220 (28.34)	592,885 (26.81)	56,150 (28.11)	70,220 (27.58)	74,160 (22.68)	141,365 (20.98)	7,870 (21.62)	7,685 (20.43)
Obese	68,440 (26.21)	163,955 (30.59)	3,575 (30.28)	7,240 (34.10)	312,095 (29.56)	240,280 (31.79)	28,840 (31.04)	19,070 (34.12)	410,370 (28.91)	697,385 (31.53)	61,520 (30.80)	85,360 (33.53)	75,910 (23.22)	166,230 (24.67)	8,330 (22.89)	9,590 (25.50)
Unknown	64,910 (24.86)	112,615 (21.01)	2,400 (20.32)	3,585 (16.88)	156,335 (14.81)	140,235 (18.55)	15,015 (16.16)	8,600 (15.39)	230,550 (16.24)	422,195 (19.09)	32,945 (16.50)	40,135 (15.76)	93,435 (28.58)	206,725 (30.68)	10,810 (29.71)	11,225 (29.84)
Ethnicity, N (%)																
White	187,630 (71.85)	387,015 (72.21)	8,780 (74.35)	16,250 (76.55)	853,175 (80.81)	593,180 (78.47)	74,780 (80.49)	46,160 (82.61)	1,139,130 (80.25)	1,763,035 (79.71)	163,125 (81.67)	214,655 (84.31)	229,930 (70.32)	479,070 (71.10)	27,475 (75.51)	28,080 (74.64)
Asian	18,025 (6.90)	53,420 (9.97)	870 (7.38)	2,065 (9.72)	39,395 (3.73)	45,590 (6.03)	4,070 (4.38)	2,785 (4.98)	59,955 (4.22)	119,025 (5.38)	9,290 (4.65)	11,290 (4.43)	27,280 (8.34)	47,755 (7.09)	2,490 (6.85)	2,440 (6.49)
Black	4,260 (1.63)	8,725 (1.63)	185 (1.58)	260 (1.23)	10,810 (1.02)	9,040 (1.20)	1,170 (1.26)	540 (0.97)	16,450 (1.16)	23,500 (1.06)	2,390 (1.20)	2,035 (0.80)	6,285 (1.92)	10,595 (1.57)	600 (1.65)	525 (1.39)
Mixed	2,855 (1.09)	5,265 (0.98)	130 (1.08)	160 (0.76)	7,120 (0.67)	6,045 (0.80)	640 (0.69)	380 (0.68)	10,410 (0.73)	17,155 (0.78)	1,530 (0.77)	1,755 (0.69)	4,035 (1.23)	8,470 (1.26)	445 (1.23)	475 (1.26)
Other	5,365 (2.05)	9,630 (1.80)	235 (1.97)	355 (1.68)	12,210 (1.16)	9,735 (1.29)	1,155 (1.24)	680 (1.22)	18,895 (1.33)	26,210 (1.19)	2,770 (1.39)	2,695 (1.06)	7,255 (2.22)	12,545 (1.86)	735 (2.02)	705 (1.87)
Unknown	42,995 (16.46)	71,885 (13.41)	1,610 (13.63)	2,135 (10.06)	133,090 (12.61)	92,360 (12.22)	11,095 (11.94)	5,335 (9.54)	174,600 (12.30)	262,760 (11.88)	20,640 (10.33)	22,170 (8.71)	52,180 (15.96)	115,370 (17.12)	4,640 (12.75)	5,395 (14.35)

CCI[3], N (%)																
Very low (=0)	182,955 (70.06)	325,880 (60.81)	7,365 (62.36)	11,495 (54.15)	574,700 (54.43)	413,945 (54.76)	51,540 (55.48)	27,285 (48.83)	780,005 (54.95)	1,198,220 (54.18)	104,310 (52.22)	119,530 (46.95)	250,480 (76.61)	530,755 (78.77)	28,035 (77.05)	29,285 (77.84)
Low (=1 and =2)	64,265 (24.61)	169,090 (31.55)	3,525 (29.83)	7,535 (35.50)	377,690 (35.77)	270,355 (35.76)	31,815 (34.24)	21,665 (38.77)	495,555 (34.91)	794,260 (35.91)	71,305 (35.70)	101,360 (39.81)	65,405 (20.00)	126,455 (18.77)	7,275 (20.00)	7,455 (19.81)
Medium (=3 and =4)	11,140 (4.27)	32,995 (6.16)	710 (6.03)	1,780 (8.39)	83,515 (7.91)	57,465 (7.60)	7,590 (8.17)	5,455 (9.76)	114,520 (8.07)	174,725 (7.90)	18,700 (9.36)	26,310 (10.33)	8,845 (2.71)	13,365 (1.98)	835 (2.30)	680 (1.81)
High (=5 and =6)	2,145 (0.82)	6,115 (1.14)	165 (1.40)	330 (1.55)	15,785 (1.49)	10,990 (1.45)	1,505 (1.62)	1,150 (2.06)	22,810 (1.61)	34,240 (1.55)	4,085 (2.05)	5,680 (2.23)	1,695 (0.52)	2,465 (0.37)	190 (0.52)	145 (0.38)
Very high (≥7)	620 (0.24)	1,855 (0.35)	45 (0.39)	85 (0.40)	4,110 (0.39)	3,195 (0.42)	455 (0.49)	325 (0.58)	6,555 (0.46)	10,245 (0.46)	1,340 (0.67)	1,720 (0.67)	550 (0.17)	755 (0.11)	45 (0.13)	55 (0.15)
Smoking status, N (%)																
Smoker	39,815 (15.25)	92,195 (17.20)	1,580 (13.38)	3,415 (16.09)	245,770 (23.28)	153,995 (20.37)	15,610 (16.80)	10,770 (19.27)	310,885 (21.90)	427,670 (19.34)	32,865 (16.45)	45,810 (17.99)	52,360 (16.01)	123,130 (18.27)	6,285 (17.27)	6,630 (17.63)
Ex-smoker	104,145 (39.88)	228,435 (42.62)	5,210 (44.09)	9,735 (45.87)	536,660 (50.83)	354,335 (46.87)	46,240 (49.77)	27,780 (49.71)	707,820 (49.87)	1,045,495 (47.27)	100,470 (50.30)	130,445 (51.24)	118,110 (36.12)	233,300 (34.62)	12,885 (35.41)	12,910 (34.32)
Never smoked	114,970 (44.03)	212,870 (39.72)	4,980 (42.14)	8,040 (37.88)	270,710 (25.64)	245,465 (32.47)	30,905 (33.27)	17,250 (30.87)	396,150 (27.91)	731,980 (33.10)	66,045 (33.06)	77,985 (30.63)	151,220 (46.25)	303,740 (45.08)	16,605 (45.64)	17,375 (46.19)
Unknown	2,200 (0.84)	2,440 (0.46)	45 (0.38)	35 (0.16)	2,660 (0.25)	2,150 (0.28)	145 (0.16)	80 (0.14)	4,595 (0.32)	6,545 (0.30)	360 (0.18)	360 (0.14)	5,280 (1.61)	13,625 (2.02)	610 (1.68)	695 (1.85)
IMD[4], N (%)																
1 (most deprived)	45,215 (17.31)	79,390 (14.81)	2,165 (18.31)	2,990 (14.08)	159,765 (15.13)	112,415 (14.87)	16,430 (17.68)	8,645 (15.47)	217,000 (15.29)	344,490 (15.58)	34,010 (17.03)	39,780 (15.62)	53,775 (16.45)	106,570 (15.82)	6,485 (17.82)	6,200 (16.49)
2	49,890 (19.10)	91,610 (17.09)	2,240 (18.96)	3,440 (16.21)	186,205 (17.64)	131,435 (17.39)	17,475 (18.81)	9,530 (17.05)	251,880 (17.75)	401,945 (18.17)	37,320 (18.69)	46,490 (18.26)	58,495 (17.89)	121,945 (18.10)	6,805 (18.70)	7,010 (18.63)
3	54,320 (20.80)	106,230 (19.82)	2,400 (20.31)	4,200 (19.79)	215,430 (20.40)	151,605 (20.06)	19,775 (21.28)	11,840 (21.19)	290,055 (20.43)	444,695 (20.11)	41,960 (21.01)	51,860 (20.37)	64,805 (19.82)	134,715 (19.99)	7,415 (20.39)	7,735 (20.56)
4	51,790 (19.83)	116,670 (21.77)	2,345 (19.85)	4,665 (21.99)	211,855 (20.07)	160,100 (21.18)	17,415 (18.75)	11,370 (20.35)	285,640 (20.12)	451,890 (20.43)	38,510 (19.28)	51,155 (20.09)	66,255 (20.26)	140,470 (20.85)	7,180 (19.74)	7,695 (20.45)
5 (most affluent)	56,035 (21.46)	133,305 (24.87)	2,485 (21.03)	5,565 (26.22)	265,170 (25.12)	188,525 (24.94)	20,365 (21.92)	13,555 (24.25)	351,615 (24.77)	532,665 (24.08)	44,695 (22.38)	60,950 (23.94)	78,115 (23.89)	159,315 (23.64)	7,825 (21.51)	8,350 (22.20)
Unknown	3,880 (1.49)	8,730 (1.63)	180 (1.53)	365 (1.72)	17,375 (1.65)	11,865 (1.57)	1,445 (1.56)	940 (1.68)	23,250 (1.64)	36,005 (1.63)	3,250 (1.63)	4,365 (1.71)	5,525 (1.69)	10,780 (1.60)	670 (1.85)	625 (1.66)
Season, N (%)																
Spring	58,730 (22.49)	121,405 (22.65)	2,840 (24.05)	5,030 (23.70)	251,725 (23.84)	171,315 (22.66)	23,785 (25.60)	13,220 (23.66)	360,205 (25.38)	507,860 (22.96)	54,005 (27.04)	62,490 (24.54)	86,600 (26.49)	177,715 (26.37)	10,325 (28.38)	10,680 (28.39)
Summer	30,840 (11.81)	69,800 (13.02)	1,365 (11.55)	2,545 (11.99)	225,995 (21.41)	121,145 (16.03)	19,630 (21.13)	9,140 (16.36)	261,695 (18.44)	308,145 (13.93)	33,040 (16.54)	34,665 (13.61)	62,290 (19.05)	127,515 (18.92)	7,550 (20.75)	7,180 (19.09)
Autumn	59,470 (22.77)	124,805 (23.29)	2,430 (20.58)	4,335 (20.43)	271,915 (25.75)	199,550 (26.40)	22,535 (24.26)	13,780 (24.66)	325,190 (22.91)	526,405 (23.80)	42,045 (21.05)	55,045 (21.62)	70,405 (21.53)	141,970 (21.07)	7,565 (20.80)	7,305 (19.42)
Winter	112,090 (42.93)	219,935 (41.04)	5,175 (43.82)	9,315 (43.88)	306,165 (29.00)	263,940 (34.91)	26,950 (29.01)	19,740 (35.32)	472,355 (33.28)	869,285 (39.30)	70,645 (35.37)	102,400 (40.22)	107,675 (32.93)	226,600 (33.63)	10,945 (30.08)	12,450 (33.10)
Region, N (%)																
London	15,875 (6.08)	39,585 (7.39)	745 (6.32)	1,185 (5.59)	30,365 (2.88)	33,805 (4.47)	3,080 (3.31)	1,735 (3.11)	53,355 (3.76)	77,460 (3.50)	9,210 (4.61)	7,760 (3.05)	18,030 (5.51)	33,785 (5.01)	1,770 (4.87)	1,570 (4.17)
North East	12,185 (4.67)	21,615 (4.03)	445 (3.78)	795 (3.74)	72,905 (6.91)	40,675 (5.38)	5,465 (5.88)	3,085 (5.52)	95,250 (6.71)	118,260 (5.35)	12,225 (6.12)	14,015 (5.50)	17,790 (5.44)	32,630 (4.84)	1,800 (4.95)	1,675 (4.46)
North West	29,655 (11.36)	47,485 (8.86)	1,195 (10.10)	1,955 (9.21)	133,295 (12.63)	82,465 (10.91)	11,910 (12.82)	6,915 (12.37)	172,490 (12.15)	248,890 (11.25)	25,105 (12.57)	32,680 (12.84)	35,855 (10.97)	66,860 (9.92)	3,800 (10.44)	3,975 (10.57)
East	56,225 (21.53)	143,295 (26.74)	2,410 (20.39)	5,155 (24.29)	224,765 (21.29)	185,845 (24.58)	19,325 (20.80)	13,155 (23.54)	304,080 (21.42)	513,490 (23.22)	41,565 (20.81)	56,270 (22.10)	75,585 (23.12)	171,975 (25.52)	8,245 (22.67)	10,085 (26.80)
West Midlands	12,075 (4.62)	32,895 (6.14)	630 (5.33)	1,350 (6.35)	38,120 (3.61)	38,340 (5.07)	3,775 (4.06)	2,540 (4.54)	53,410 (3.76)	107,780 (4.87)	7,950 (3.98)	10,955 (4.30)	16,670 (5.10)	34,100 (5.06)	1,650 (4.54)	1,845 (4.90)
Yorkshire and The Humber	40,060 (15.34)	101,440 (18.93)	2,630 (22.28)	4,285 (20.18)	173,795 (16.46)	117,625 (15.56)	16,105 (17.34)	8,805 (15.76)	231,640 (16.32)	389,705 (17.62)	34,800 (17.42)	45,745 (17.97)	51,465 (15.74)	110,675 (16.43)	6,215 (17.08)	5,800 (15.42)

South East	17,195 (6.59)	25,795 (4.81)	680 (5.74)	1,195 (5.63)	64,540 (6.11)	46,340 (6.13)	5,785 (6.23)	3,785 (6.77)	86,705 (6.11)	124,800 (5.64)	11,600 (5.81)	14,135 (5.55)	19,315 (5.91)	35,815 (5.32)	2,315 (6.37)	2,145 (5.70)
East Midlands	49,750 (19.05)	88,095 (16.44)	2,145 (18.15)	4,230 (19.94)	185,820 (17.60)	134,425 (17.78)	17,095 (18.40)	10,200 (18.25)	247,100 (17.41)	413,650 (18.70)	36,505 (18.28)	50,305 (19.76)	58,700 (17.95)	124,000 (18.40)	6,580 (18.08)	7,060 (18.77)
South West	28,110 (10.76)	35,730 (6.67)	935 (7.91)	1,080 (5.08)	132,195 (12.52)	76,430 (10.11)	10,360 (11.15)	5,660 (10.13)	175,415 (12.36)	217,660 (9.84)	20,780 (10.40)	22,740 (8.93)	33,565 (10.27)	63,955 (9.49)	4,005 (11.00)	3,460 (9.20)
Flu vaccination, N (%)																
Yes	80,510 (30.83)	212,950 (39.73)	4,585 (38.83)	9,975 (46.99)	570,140 (54.00)	355,710 (47.05)	46,935 (50.52)	29,775 (53.29)	734,940 (51.78)	1,053,160 (47.62)	103,365 (51.75)	141,080 (55.41)	72,575 (22.20)	118,375 (17.57)	7,335 (20.16)	6,645 (17.66)
No	180,620 (69.17)	322,990 (60.27)	7,225 (61.17)	11,255 (53.01)	485,660 (46.00)	400,240 (52.95)	45,965 (49.48)	26,105 (46.71)	684,505 (48.22)	1,158,530 (52.38)	96,370 (48.25)	113,520 (44.59)	254,395 (77.80)	555,420 (82.43)	29,050 (79.84)	30,975 (82.34)
Period																
Pre-pandemic	123,595 (47.33)	192,575 (35.93)	5,410 (45.82)	8,520 (40.14)	338,250 (32.04)	252,410 (33.39)	32,685 (35.18)	20,705 (37.05)	422,410 (29.76)	758,745 (34.31)	69,005 (34.55)	96,365 (37.85)	121,335 (37.11)	223,405 (33.16)	12,610 (34.65)	13,095 (34.82)
During pandemic	12,195 (4.67)	33,965 (6.34)	755 (6.37)	1,325 (6.25)	174,660 (16.54)	82,210 (10.88)	16,325 (17.57)	7,140 (12.78)	201,700 (14.21)	178,585 (8.07)	24,880 (12.46)	20,860 (8.19)	38,915 (11.90)	73,715 (10.94)	5,865 (16.12)	4,425 (11.77)
After 2nd lockdown	90,005 (34.47)	254,410 (47.47)	3,855 (32.65)	8,575 (40.41)	542,890 (51.42)	421,330 (55.74)	43,895 (47.25)	28,035 (50.17)	646,080 (45.52)	1,050,820 (47.51)	79,960 (40.03)	103,920 (40.82)	128,835 (39.40)	305,845 (45.39)	13,875 (38.13)	15,355 (40.82)
Count of antibiotic prescription, mean (SD[5])	1.01 (1.62)	2.33 (1.79)	2.06 (1.99)	3.59 (2.00)	1.59 (2.30)	2.75 (2.36)	2.27 (2.44)	3.95 (2.64)	1.55 (2.29)	2.68 (2.28)	2.59 (2.52)	4.11 (2.57)	0.88 (1.34)	1.9 (1.26)	1.7 (1.51)	2.78 (1.38)

[1] ABs, antibiotics prescribed or not.
[2] BMI, Body Mass Index recorded in the last 5 years.
[3] CCI, Charlson Comorbidities Index, measured from 17 weighted conditions, including myocardial infarction, congestive heart failure, peripheral vascular disease, cerebrovascular disease, dementia, chronic pulmonary disease, Connective tissue disease, ulcer disease, mild liver disease, diabetes, hemiplegia, moderate or severe renal disease, diabetes with complications, any malignancy (including leukaemia and lymphoma), moderate or severe liver disease, metastatic solid tumour, and AIDS.
[4] IMD, Multiple Deprivation Index, quintile measured from patient-level address.
[5] SD, standard deviation.

Counts and rates of hospital admission cases

Table S4. Counts and observed rates of infection-related hospital admission in deciles of predicted probability of hospital admission related to prevalent common infections, including lower respiratory tract infection (LRTI), upper respiratory tract infection (URTI), and urinary tract infection (UTI), using data from January 2019 to March 2023.

Deciles[1] of predicted risk	Prevalent LRTI N cases (Observed rate[2] of hospital admissions)	Prevalent URTI N cases (Observed rate of hospital admissions)	Prevalent UTI N cases (Observed rate of hospital admissions)
Decile 1 (lowest)	325 (11.7)	315 (4.3)	270 (6.7)
Decile 2	535 (19.3)	735 (10.1)	660 (16.3)
Decile 3	560 (20.2)	915 (12.5)	730 (18.1)
Decile 4	685 (24.7)	1,105 (15.2)	880 (21.8)
Decile 5	805 (29.1)	1,330 (18.2)	1,190 (29.4)
Decile 6	1,080 (39.0)	1,525 (20.9)	1,585 (39.2)
Decile 7	1,425 (51.6)	1,920 (26.3)	2,060 (50.9)
Decile 8	1,820 (65.7)	2,550 (34.9)	2,590 (64.0)
Decile 9	2,150 (77.7)	3,635 (49.8)	3,135 (77.5)
Decile 10 (highest)	2,720 (98.4)	5,425 (74.3)	4,175 (103.2)

[1] Deciles are calculated by grouping predicted probability of infection-related hospital admission using Cox models
[2] Rate is the number of cases per 1000 patients with common infection, calculated by dividing the count of infection-related hospital admission cases (numerator) by the count of infection diagnosis (denominator) and then multiplied by 1000

Table S5. Counts and rates of infection-related hospital admission in deciles of predicted probability of hospital admission related to other common infections, including sinusitis, otitis media, and otitis externa, using data from January 2019 to March 2023.

Deciles[1] of predicted risk	Sinusitis N cases (Observed rate[2] of hospital admissions)	Otitis media N cases (Observed rate of hospital admissions)		Otitis externa N cases (Observed rate of hospital admissions)	
	Incident	Incident	Prevalent	Incident	Prevalent
Decile 1 (lowest)	30 (0.5)	45 (1.2)	10 (3.5)	65 (0.7)	20 (2.3)
Decile 2	80 (1.3)	110 (2.9)	20 (5.8)	165 (1.8)	60 (6.3)
Decile 3	100 (1.6)	165 (4.4)	15 (5.2)	255 (2.9)	60 (6.2)
Decile 4	135 (2.2)	190 (5.0)	25 (7.7)	330 (3.7)	120 (12.5)
Decile 5	150 (2.4)	205 (5.4)	25 (8.7)	390 (4.4)	95 (10.2)
Decile 6	155 (2.5)	240 (6.4)	25 (8.4)	445 (5.0)	105 (11.1)
Decile 7	200 (3.2)	290 (7.7)	35 (10.6)	525 (5.9)	160 (16.7)
Decile 8	225 (3.6)	295 (7.9)	50 (15.8)	600 (6.8)	145 (15.2)
Decile 9	315 (5.1)	385 (10.2)	55 (17.4)	885 (10.0)	310 (32.7)
Decile 10 (highest)	635 (10.3)	755 (20.1)	130 (41.9)	1,800 (20.3)	660 (69.3)

[1] Deciles are calculated by grouping predicted probability of infection-related hospital admission using Cox models

[2] Rate is the number of cases per 1000 patients with common infection, calculated by dividing the count of infection-related hospital admission cases (numerator) by the count of infection diagnosis (denominator) and then multiplied by 1000

Table S6. Counts and rates of infection-related hospital admission in deciles of predicted probability of hospital admission related to upper respiratory tract infections (URTIs), including specific URTI, cough, cold with cough, and sore throat, using data from January 2019 to March 2023.

Deciles[1] of predicted risk	Specific URTI N cases (Observed rate[2] of hospital admissions)		Cough N cases (Observed rate of hospital admissions)		Cold with cough N cases (Observed rate of hospital admissions)		Sore throat N cases (Observed rate of hospital admissions)	
	Incident	Prevalent	Incident	Prevalent	Incident	Prevalent	Incident	Prevalent
Decile 1 (lowest)	95 (1.2)	10 (3.6)	270 (1.5)	45 (3.0)	930 (2.6)	240 (5.3)	200 (2.0)	50 (7.0)
Decile 2	280 (3.5)	15 (5.1)	825 (4.5)	75 (5.1)	2,550 (7.0)	545 (12.0)	460 (4.6)	85 (11.2)
Decile 3	420 (5.3)	30 (8.8)	1,040 (5.7)	90 (6.1)	3,035 (8.4)	640 (14.0)	670 (6.7)	100 (13.6)
Decile 4	475 (6.0)	40 (12.7)	1,215 (6.7)	155 (10.4)	3,490 (9.6)	705 (15.5)	780 (7.8)	120 (15.9)
Decile 5	560 (7.1)	40 (12.7)	1,350 (7.5)	150 (10.0)	4,105 (11.3)	880 (19.3)	920 (9.2)	155 (20.8)
Decile 6	650 (8.2)	40 (12.1)	1,620 (8.9)	190 (12.7)	5,010 (13.8)	1,090 (24.0)	1,035 (10.3)	190 (25.4)
Decile 7	815 (10.2)	50 (15.1)	2,095 (11.6)	250 (16.7)	6,385 (17.6)	1,480 (32.6)	1,225 (12.3)	200 (27.0)
Decile 8	1,145 (14.4)	80 (23.9)	2,840 (15.7)	355 (23.9)	9,420 (25.9)	2,070 (45.5)	1,400 (14.0)	200 (27.3)
Decile 9	1,875 (23.5)	115 (34.5)	4,015 (22.2)	500 (33.5)	14,255 (39.3)	2,775 (61.0)	1,680 (16.8)	275 (37.4)
Decile 10 (highest)	3,675 (46.1)	230 (69.0)	7,195 (39.7)	750 (50.5)	22,280 (61.4)	3,755 (82.6)	2,265 (22.6)	375 (50.9)

[1] Deciles are calculated by grouping predicted probability of infection-related hospital admission using Cox models

[2] Rate is the number of cases per 1000 patients with common infection, calculated by dividing the count of infection-related hospital admission cases (numerator) by the count of infection diagnosis (denominator) and then multiplied by 1000

Logistic regression models

Performance

Table S7. Area under the receiver operating characteristic curve of logistic regression models for prescribing antibiotics with deciles of predicted risk of hospital admissions related to common infections, namely lower respiratory tract infection (LRTI), upper respiratory tract infection (URTI), urinary tract infections (UTI), sinusitis, otitis media, otitis externa and components of URTI including specific URTI, cough, cold with cough, and sore throat, using data from January 2019 to March 2022.

Infection	Type	AUROC[1]	
		Development dataset	Validation dataset
LRTI	Prevalent	0.53	0.53
URTI	Prevalent	0.59	0.59
UTI	Prevalent	0.52	0.52
Sinusitis	Incident	0.60	0.60
	Prevalent	-	-
Otitis media	Incident	0.57	0.57
	Prevalent	0.53	0.55
Otitis externa	Incident	0.61	0.61
	Prevalent	0.56	0.55
URTI components			
Specific URTI	Incident	0.64	0.64
	Prevalent	0.62	0.63
Cough	Incident	0.56	0.56
	Prevalent	0.60	0.60
Cold with cough	Incident	0.55	0.55
	Prevalent	0.58	0.58
Sore throat	Incident	0.56	0.56
	Prevalent	0.56	0.56

[1]AUROC, area under the receiver operating characteristic curve.

Odds ratios

Table S8. Adjusted odds ratios of being prescribed an antibiotic for incident and prevalent lower respiratory tract infection (LRTI), upper respiratory tract infection (URTI), urinary tract infection (UTI), using data from January 2019 to March 2023, with deciles of predicted probability of hospital admission related to the infections.

	LRTI Adjusted OR[1] (95% CI[2])		URTI Adjusted OR[1] (95% CI[2])		UTI Adjusted OR[1] (95% CI[2])	
	Incident	Prevalent	Incident	Prevalent	Incident	Prevalent
Decile 1 (lowest)	Reference	Reference	Reference	Reference	Reference	Reference
Decile 2	0.89 (0.87-0.91)	0.95 (0.91-0.99)	0.77 (0.76-0.77)	0.84 (0.82-0.86)	0.93 (0.91-0.95)	1.02 (0.98-1.06)
Decile 3	0.95 (0.93-0.97)	1.14 (1.09-1.20)	0.92 (0.92-0.93)	1.09 (1.07-1.12)	0.98 (0.96-1.00)	1.08 (1.04-1.12)
Decile 4	0.97 (0.95-0.99)	1.30 (1.25-1.36)	1.09 (1.08-1.10)	1.32 (1.29-1.35)	1.06 (1.04-1.09)	1.15 (1.11-1.20)
Decile 5	0.96 (0.94-0.98)	1.33 (1.27-1.39)	1.19 (1.18-1.19)	1.53 (1.49-1.57)	1.09 (1.07-1.12)	1.15 (1.11-1.20)
Decile 6	0.91 (0.89-0.93)	1.22 (1.17-1.27)	1.31 (1.30-1.31)	1.71 (1.67-1.75)	1.10 (1.08-1.12)	1.13 (1.09-1.17)
Decile 7	0.80 (0.79-0.82)	1.07 (1.03-1.12)	1.44 (1.42-1.45)	1.81 (1.76-1.86)	1.02 (1.00-1.04)	1.05 (1.01-1.09)
Decile 8	0.66 (0.65-0.68)	1.03 (0.98-1.08)	1.35 (1.34-1.36)	1.91 (1.86-1.96)	0.90 (0.88-0.92)	1.04 (1.00-1.08)
Decile 9	0.56 (0.55-0.58)	1.02 (0.98-1.07)	1.12 (1.12-1.13)	2.04 (1.99-2.09)	0.80 (0.78-0.81)	0.96 (0.92-0.99)
Decile 10 (highest)	0.52 (0.51-0.54)	1.13 (1.08-1.18)	1.42 (1.41-1.43)	2.42 (2.35-2.49)	0.65 (0.64-0.66)	0.88 (0.85-0.91)

[1] OR, odds ratio.
[2] CI, confidence interval.

Table S9. Adjusted odds ratios of being prescribed an antibiotic for sinusitis, otitis externa, and otitis media, using data from January 2019 to March 2023, with deciles of predicted probability of hospital admission related to the infections.

	Sinusitis Adjusted OR[1] (95% CI[2])	URTI Adjusted OR[1] (95% CI[2])		UTI Adjusted OR[1] (95% CI[2])	
	Incident	Incident	Prevalent	Incident	Prevalent
Decile 1 (lowest)	Reference	Reference	Reference	Reference	Reference
Decile 2	0.70 (0.69-0.72)	1.37 (1.33-1.42)	1.01 (0.94-1.09)	0.77 (0.74-0.79)	1.13 (1.02-1.24)
Decile 3	0.81 (0.78-0.83)	0.91 (0.88-0.94)	1.04 (0.96-1.12)	0.84 (0.81-0.87)	1.17 (1.04-1.31)
Decile 4	1.08 (1.04-1.11)	1.12 (1.09-1.16)	1.11 (1.03-1.21)	1.01 (0.98-1.05)	1.28 (1.14-1.44)
Decile 5	1.30 (1.26-1.34)	1.44 (1.39-1.48)	1.35 (1.25-1.45)	1.15 (1.11-1.19)	1.31 (1.17-1.47)
Decile 6	1.52 (1.48-1.56)	1.82 (1.77-1.88)	1.46 (1.36-1.57)	1.30 (1.25-1.35)	1.30 (1.15-1.46)
Decile 7	1.74 (1.69-1.80)	2.23 (2.17-2.29)	1.58 (1.47-1.70)	1.43 (1.38-1.49)	1.44 (1.27-1.63)
Decile 8	1.81 (1.76-1.87)	2.53 (2.46-2.59)	1.77 (1.65-1.91)	1.54 (1.49-1.59)	1.40 (1.23-1.58)
Decile 9	1.81 (1.75-1.87)	2.59 (2.51-2.68)	1.69 (1.57-1.82)	1.53 (1.48-1.59)	1.55 (1.39-1.73)
Decile 10 (highest)	1.98 (1.92-2.04)	2.67 (2.59-2.75)	1.53 (1.42-1.66)	1.45 (1.40-1.51)	1.30 (1.16-1.47)

[1] OR, odds ratio.
[2] CI, confidence interval.

Table S10. Adjusted odds ratios of being prescribed an antibiotic for specific upper respiratory tract infection (URTI), cough, cold with cough, and sore throat, using data from January 2019 to March 2023, with deciles of predicted probability of hospital admission related to the infections.

	Specific URTI Adjusted OR[1] (95% CI[2])		Cough Adjusted OR[1] (95% CI[2])		Cold with cough Adjusted OR[1] (95% CI[2])		Sore throat Adjusted OR[1] (95% CI[2])	
	Incident	Prevalent	Incident	Prevalent	Incident	Prevalent	Incident	Prevalent
Decile 1 (lowest)	Reference	Reference	Reference	Reference	Reference	Reference	Reference	Reference
Decile 2	0.73 (0.71-0.74)	0.84 (0.75-0.94)	0.77 (0.76-0.78)	1.08 (1.01-1.15)	0.92 (0.91-0.93)	0.94 (0.91-0.96)	0.92 (0.90-0.94)	1.05 (0.97-1.15)
Decile 3	0.90 (0.88-0.92)	1.03 (0.92-1.16)	0.89 (0.87-0.90)	1.26 (1.19-1.34)	1.04 (1.03-1.05)	1.18 (1.15-1.22)	0.87 (0.86-0.89)	1.03 (0.95-1.12)
Decile 4	1.27 (1.25-1.30)	1.46 (1.31-1.63)	1.06 (1.04-1.07)	1.38 (1.30-1.45)	1.15 (1.14-1.16)	1.38 (1.34-1.41)	1.06 (1.03-1.08)	1.16 (1.08-1.26)
Decile 5	1.65 (1.61-1.68)	1.64 (1.46-1.84)	1.22 (1.20-1.24)	1.59 (1.50-1.68)	1.27 (1.25-1.28)	1.60 (1.56-1.65)	1.16 (1.13-1.18)	1.31 (1.22-1.41)
Decile 6	2.03 (1.98-2.08)	1.99 (1.76-2.24)	1.35 (1.33-1.37)	1.71 (1.62-1.80)	1.35 (1.33-1.36)	1.75 (1.69-1.80)	1.23 (1.20-1.25)	1.38 (1.28-1.48)
Decile 7	2.40 (2.35-2.46)	1.98 (1.78-2.21)	1.39 (1.37-1.41)	1.90 (1.79-2.01)	1.42 (1.40-1.44)	1.77 (1.71-1.83)	1.32 (1.29-1.35)	1.50 (1.39-1.63)
Decile 8	2.65 (2.58-2.73)	2.12 (1.89-2.38)	1.29 (1.27-1.31)	2.12 (2.00-2.25)	1.27 (1.26-1.28)	1.76 (1.72-1.81)	1.46 (1.43-1.49)	1.62 (1.50-1.76)
Decile 9	2.78 (2.71-2.85)	2.54 (2.25-2.88)	1.32 (1.30-1.34)	2.53 (2.40-2.66)	1.22 (1.21-1.24)	1.86 (1.81-1.92)	1.52 (1.49-1.55)	1.58 (1.47-1.70)
Decile 10 (highest)	3.52 (3.44-3.61)	3.25 (2.85-3.70)	1.63 (1.60-1.65)	3.07 (2.92-3.24)	1.61 (1.60-1.63)	2.31 (2.24-2.38)	1.71 (1.66-1.75)	1.83 (1.69-1.98)

[1] OR, odds ratio.
[2] CI, confidence interval.

Table S11. Adjusted odds ratios of being prescribed an antibiotic for incident and prevalent lower respiratory tract infection (LRTI), upper respiratory tract infection (URTI), urinary tract infection (UTI), using data from January 2019 to March 2023, with risk factors as covariates.

	LRTI Adjusted OR[1] (95% CI[2])		URTI Adjusted OR[1] (95% CI[2])		UTI Adjusted OR[1] (95% CI[2])	
	Incident	Prevalent	Incident	Prevalent	Incident	Prevalent
**Sex**						
Male	0.92 (0.91-0.93)	1.05 (1.03-1.07)	0.92 (0.91-0.92)	1.00 (0.99-1.01)	0.54 (0.53-0.54)	0.65 (0.63-0.66)
**Age**						
25-34	1.14 (1.11-1.17)	1.14 (1.07-1.22)	1.09 (1.08-1.10)	1.04 (1.01-1.07)	1.25 (1.23-1.27)	1.13 (1.07-1.18)
35-44	1.23 (1.20-1.26)	1.20 (1.12-1.29)	1.02 (1.02-1.03)	0.99 (0.96-1.03)	1.45 (1.42-1.47)	1.30 (1.23-1.37)
45-54	1.22 (1.19-1.25)	1.25 (1.17-1.33)	0.89 (0.88-0.89)	0.91 (0.89-0.94)	1.52 (1.49-1.55)	1.39 (1.32-1.46)
55-64	1.17 (1.14-1.20)	1.29 (1.21-1.37)	0.76 (0.75-0.77)	0.89 (0.86-0.91)	1.57 (1.54-1.60)	1.38 (1.31-1.45)
65-74	0.94 (0.92-0.97)	1.20 (1.13-1.28)	0.66 (0.65-0.66)	0.86 (0.84-0.89)	1.49 (1.47-1.52)	1.37 (1.31-1.43)
75+	0.68 (0.67-0.70)	0.93 (0.87-0.99)	0.69 (0.69-0.70)	0.87 (0.85-0.90)	1.11 (1.09-1.13)	1.10 (1.05-1.15)
**BMI[3]**						
Underweight	0.71 (0.69-0.73)	0.81 (0.77-0.86)	0.82 (0.81-0.83)	0.95 (0.91-1.00)	0.85 (0.82-0.88)	0.87 (0.82-0.93)
Overweight	1.25 (1.23-1.26)	1.21 (1.18-1.25)	1.13 (1.13-1.14)	1.08 (1.07-1.10)	1.05 (1.04-1.06)	1.05 (1.03-1.08)
Obese	1.29 (1.27-1.30)	1.29 (1.26-1.33)	1.21 (1.20-1.21)	1.12 (1.10-1.14)	1.00 (0.99-1.02)	1.01 (0.98-1.04)
Unknown	1.13 (1.12-1.15)	1.14 (1.11-1.18)	1.35 (1.34-1.36)	1.16 (1.14-1.19)	1.01 (0.99-1.02)	1.02 (0.99-1.04)
**Ethnicity**						
Asian	1.25 (1.22-1.27)	1.11 (1.06-1.18)	1.16 (1.15-1.17)	1.05 (1.02-1.08)	1.05 (1.03-1.08)	1.10 (1.05-1.17)
Black	0.89 (0.85-0.92)	0.89 (0.80-0.98)	0.93 (0.92-0.94)	0.84 (0.80-0.89)	0.98 (0.95-1.02)	0.99 (0.88-1.11)
Mixed	1.01 (0.96-1.06)	1.02 (0.90-1.16)	1.01 (0.99-1.03)	0.97 (0.91-1.02)	0.96 (0.92-1.00)	1.04 (0.94-1.16)
Other	0.96 (0.92-0.99)	1.02 (0.92-1.13)	0.93 (0.91-0.94)	1.00 (0.96-1.05)	0.91 (0.88-0.94)	0.91 (0.83-1.00)
Unknown	1.65 (1.63-1.68)	1.22 (1.17-1.26)	1.09 (1.08-1.09)	1.09 (1.07-1.11)	1.46 (1.44-1.49)	1.24 (1.20-1.28)
**CCI[4]**						
Low	0.85 (0.85-0.86)	0.85 (0.83-0.87)	0.95 (0.95-0.96)	0.97 (0.96-0.98)	0.88 (0.87-0.89)	0.86 (0.84-0.88)
Medium	0.73 (0.72-0.74)	0.74 (0.72-0.76)	0.92 (0.91-0.92)	0.93 (0.91-0.96)	0.75 (0.74-0.76)	0.77 (0.74-0.79)
High	0.61 (0.59-0.63)	0.63 (0.59-0.67)	0.86 (0.85-0.88)	0.83 (0.80-0.87)	0.64 (0.62-0.66)	0.67 (0.63-0.71)
Very high	0.52 (0.49-0.54)	0.54 (0.49-0.60)	0.89 (0.86-0.91)	0.81 (0.75-0.86)	0.55 (0.52-0.57)	0.62 (0.57-0.69)
**Smoking status**						
Smoker	1.08 (1.07-1.09)	1.14 (1.11-1.18)	0.89 (0.88-0.89)	1.03 (1.01-1.04)	0.97 (0.96-0.99)	0.96 (0.93-0.99)
Never smoked	1.03 (1.02-1.04)	0.99 (0.97-1.02)	1.21 (1.21-1.22)	1.05 (1.04-1.06)	1.00 (0.99-1.01)	0.97 (0.96-0.99)
Unknown	0.81 (0.76-0.87)	0.74 (0.58-0.95)	1.11 (1.08-1.13)	1.13 (1.01-1.25)	0.87 (0.81-0.93)	0.84 (0.72-0.97)
**IMD[5]**						

1 (most deprived)	0.98 (0.97-1.00)	0.93 (0.90-0.96)	1.01 (1.00-1.01)	0.97 (0.95-0.99)	1.01 (1.00-1.03)	0.97 (0.95-1.00)
3	0.98 (0.96-0.99)	0.99 (0.97-1.02)	0.95 (0.95-0.96)	1.00 (0.98-1.02)	0.97 (0.96-0.98)	0.99 (0.96-1.02)
4	0.98 (0.96-0.99)	1.04 (1.01-1.08)	0.96 (0.96-0.97)	1.02 (1.01-1.04)	0.93 (0.91-0.94)	0.97 (0.95-1.00)
5 (most affluent)	0.91 (0.90-0.92)	1.07 (1.04-1.11)	0.89 (0.88-0.89)	0.99 (0.97-1.01)	0.88 (0.86-0.89)	0.98 (0.95-1.01)
Unknown	0.95 (0.92-0.99)	1.11 (1.02-1.21)	0.91 (0.89-0.92)	1.01 (0.96-1.06)	0.88 (0.85-0.91)	1.01 (0.93-1.09)
**Season**						
Spring	0.90 (0.89-0.91)	0.94 (0.91-0.97)	0.92 (0.91-0.92)	0.94 (0.92-0.95)	1.00 (0.99-1.01)	1.02 (0.99-1.05)
Summer	0.78 (0.77-0.79)	0.84 (0.81-0.88)	0.79 (0.78-0.79)	0.80 (0.79-0.82)	1.00 (0.99-1.01)	1.00 (0.97-1.03)
Winter	1.12 (1.11-1.13)	1.10 (1.07-1.14)	1.15 (1.14-1.15)	1.17 (1.15-1.18)	1.00 (0.99-1.01)	1.04 (1.02-1.07)
**Region**						
London	0.79 (0.77-0.80)	0.68 (0.63-0.73)	1.03 (1.02-1.04)	0.84 (0.82-0.87)	0.76 (0.75-0.78)	0.66 (0.62-0.70)
North East	0.87 (0.85-0.89)	0.88 (0.84-0.92)	0.76 (0.75-0.76)	0.84 (0.82-0.87)	0.83 (0.81-0.85)	0.89 (0.85-0.93)
North West	0.99 (0.98-1.01)	0.96 (0.93-1.00)	0.82 (0.82-0.83)	0.91 (0.89-0.93)	1.00 (0.98-1.02)	1.01 (0.98-1.05)
West Midlands	1.05 (1.03-1.08)	0.97 (0.92-1.02)	1.16 (1.15-1.17)	0.99 (0.96-1.02)	1.08 (1.06-1.11)	1.07 (1.01-1.13)
Yorkshire and The Humber	1.18 (1.17-1.20)	0.99 (0.96-1.02)	0.98 (0.97-0.99)	0.92 (0.90-0.93)	1.10 (1.08-1.12)	0.88 (0.86-0.91)
South East	0.87 (0.85-0.89)	0.94 (0.90-0.98)	0.85 (0.84-0.86)	0.91 (0.89-0.94)	0.88 (0.86-0.90)	0.93 (0.89-0.97)
East Midlands	1.10 (1.09-1.12)	1.10 (1.06-1.14)	0.94 (0.94-0.95)	1.00 (0.98-1.02)	1.16 (1.14-1.17)	1.11 (1.08-1.14)
South West	0.82 (0.80-0.83)	0.81 (0.78-0.84)	0.74 (0.74-0.75)	0.84 (0.83-0.86)	0.87 (0.86-0.89)	0.84 (0.82-0.86)
**Flu vaccination**						
Yes	1.02 (1.01-1.03)	1.03 (1.01-1.06)	0.81 (0.81-0.82)	0.95 (0.94-0.96)	1.06 (1.05-1.07)	1.04 (1.01-1.06)
**Count of antibiotic prescription in the one year before**	1.16 (1.15-1.16)	1.19 (1.19-1.20)	1.38 (1.38-1.38)	1.34 (1.34-1.34)	1.16 (1.16-1.16)	1.15 (1.14-1.15)

[1] OR, odds ratio.
[2] CI, confidence interval.
[3] BMI, Body Mass Index recorded in the last 5 years.
[4] CCI, Charlson Comorbidities Index, measured from 17 weighted conditions, including myocardial infarction, congestive heart failure, peripheral vascular disease, cerebrovascular disease, dementia, chronic pulmonary disease, Connective tissue disease, ulcer disease, mild liver disease, diabetes, hemiplegia, moderate or severe renal disease, diabetes with complications, any malignancy (including leukaemia and lymphoma), moderate or severe liver disease, metastatic solid tumour, and AIDS.
[5] IMD, Multiple Deprivation Index, quintile measured from patient-level address.
Reference group for variable sex is female, for age is 18-25, for BMI is healthy weight, for ethnicity is non-white, for CCI is very low, for smoking status is ex-smoker, for IMD is 2, for season is autumn, for region is east, for flu vaccination is no.

Table S12. Adjusted odds ratios of being prescribed an antibiotic for sinusitis, otitis externa, and otitis media, using data from January 2019 to March 2023, with risk factors as covariates.

	Sinusitis Adjusted OR[1] (95% CI[2])	URTI Adjusted OR[1] (95% CI[2])		UTI Adjusted OR[1] (95% CI[2])	
	Incident	Incident	Prevalent	Incident	Prevalent
**Sex**					
Male	0.81 (0.80-0.83)	1.05 (1.04-1.06)	1.07 (1.03-1.11)	0.94 (0.93-0.96)	1.06 (1.00-1.13)
**Age**					
25-34	1.22 (1.18-1.25)	1.07 (1.04-1.10)	1.20 (1.11-1.31)	1.09 (1.06-1.13)	1.10 (1.00-1.21)
35-44	1.34 (1.30-1.39)	1.10 (1.07-1.13)	1.19 (1.10-1.30)	1.12 (1.09-1.16)	1.10 (1.00-1.23)
45-54	1.37 (1.32-1.41)	1.06 (1.04-1.09)	1.14 (1.06-1.23)	1.04 (1.01-1.08)	1.02 (0.92-1.12)
55-64	1.37 (1.33-1.42)	0.96 (0.94-0.99)	1.11 (1.02-1.19)	0.94 (0.91-0.97)	1.00 (0.90-1.11)
65-74	1.28 (1.24-1.32)	0.84 (0.82-0.87)	0.98 (0.91-1.06)	0.84 (0.81-0.87)	0.92 (0.81-1.04)
75+	1.04 (1.00-1.09)	0.71 (0.69-0.74)	0.88 (0.80-0.96)	0.64 (0.61-0.67)	0.71 (0.60-0.83)
**BMI[3]**					
Underweight	0.95 (0.89-1.01)	0.89 (0.84-0.95)	0.92 (0.78-1.07)	0.94 (0.87-1.01)	1.05 (0.81-1.37)
Overweight	1.01 (1.00-1.03)	1.06 (1.04-1.08)	1.03 (0.98-1.09)	1.08 (1.05-1.11)	1.07 (0.99-1.16)
Obese	1.03 (1.01-1.05)	1.11 (1.09-1.13)	1.03 (0.98-1.08)	1.06 (1.04-1.10)	1.03 (0.96-1.12)
Unknown	1.12 (1.10-1.14)	1.15 (1.13-1.17)	1.08 (1.02-1.14)	1.10 (1.08-1.13)	1.07 (0.99-1.15)
**Ethnicity**					
Asian	0.90 (0.87-0.93)	0.87 (0.85-0.90)	0.93 (0.84-1.02)	0.98 (0.95-1.02)	0.91 (0.80-1.03)
Black	0.83 (0.77-0.90)	0.82 (0.77-0.88)	0.95 (0.79-1.13)	0.92 (0.85-1.00)	1.05 (0.76-1.44)
Mixed	0.89 (0.83-0.95)	0.90 (0.84-0.97)	0.92 (0.76-1.10)	1.01 (0.92-1.12)	1.32 (0.97-1.79)
Other	0.88 (0.84-0.93)	0.88 (0.83-0.94)	0.76 (0.67-0.87)	1.07 (0.99-1.15)	1.06 (0.87-1.29)
Unknown	1.26 (1.23-1.28)	1.11 (1.09-1.13)	1.19 (1.12-1.25)	1.34 (1.31-1.37)	1.11 (1.02-1.19)
**CCI[4]**					
Low	0.84 (0.83-0.86)	0.91 (0.90-0.93)	0.93 (0.88-0.97)	0.89 (0.87-0.91)	0.93 (0.88-0.98)
Medium	0.86 (0.82-0.91)	0.87 (0.84-0.90)	0.91 (0.84-0.99)	0.82 (0.78-0.87)	0.70 (0.61-0.81)
High	0.74 (0.67-0.83)	0.83 (0.76-0.90)	0.89 (0.75-1.06)	0.74 (0.66-0.83)	0.99 (0.73-1.33)
Very high	0.77 (0.62-0.95)	0.80 (0.69-0.92)	0.93 (0.66-1.31)	0.52 (0.43-0.65)	1.28 (0.67-2.44)
**Smoking status**					
Smoker	1.06 (1.04-1.09)	1.12 (1.10-1.14)	0.99 (0.95-1.05)	1.01 (0.99-1.04)	0.93 (0.86-1.01)
Never smoked	1.03 (1.02-1.05)	0.99 (0.97-1.00)	0.97 (0.93-1.01)	1.03 (1.01-1.05)	1.00 (0.95-1.06)
Unknown	1.14 (1.05-1.24)	1.03 (0.96-1.11)	1.05 (0.84-1.31)	1.12 (1.03-1.22)	1.25 (0.94-1.64)
**IMD[5]**					
1 (most deprived)	0.99 (0.97-1.01)	0.96 (0.94-0.98)	0.96 (0.91-1.01)	0.97 (0.94-1.00)	0.88 (0.81-0.97)
3	0.97 (0.95-1.00)	1.01 (0.99-1.03)	0.99 (0.94-1.05)	1.00 (0.97-1.03)	0.94 (0.86-1.03)
4	0.95 (0.93-0.97)	1.03 (1.01-1.05)	1.00 (0.95-1.05)	1.00 (0.97-1.04)	1.06 (0.97-1.16)
5 (most affluent)	0.88 (0.85-0.90)	1.01 (0.99-1.03)	0.96 (0.91-1.02)	0.98 (0.95-1.01)	1.07 (0.98-1.17)

Unknown	0.92 (0.87-0.98)	1.05 (1.00-1.10)	0.98 (0.84-1.13)	0.92 (0.85-0.99)	0.99 (0.81-1.22)
**Season**					
Spring	1.02 (1.00-1.04)	0.98 (0.97-1.00)	0.98 (0.93-1.03)	1.01 (0.99-1.03)	1.05 (0.96-1.15)
Summer	0.90 (0.88-0.92)	0.98 (0.97-1.00)	1.01 (0.96-1.06)	0.95 (0.92-0.98)	1.01 (0.93-1.09)
Winter	1.17 (1.15-1.19)	1.02 (1.00-1.04)	1.01 (0.96-1.05)	1.08 (1.05-1.10)	1.01 (0.93-1.09)
**Region**					
London	0.74 (0.71-0.77)	0.91 (0.88-0.94)	0.79 (0.72-0.87)	0.86 (0.83-0.90)	0.82 (0.70-0.95)
North East	0.71 (0.68-0.73)	0.85 (0.83-0.87)	0.98 (0.90-1.07)	0.87 (0.83-0.90)	0.95 (0.83-1.08)
North West	0.74 (0.73-0.76)	0.79 (0.77-0.81)	0.87 (0.81-0.92)	0.96 (0.94-0.99)	0.97 (0.88-1.08)
West Midlands	0.90 (0.87-0.93)	0.89 (0.86-0.93)	0.88 (0.79-0.97)	1.01 (0.97-1.05)	1.03 (0.89-1.20)
Yorkshire and The Humber	0.80 (0.79-0.82)	0.83 (0.82-0.85)	0.88 (0.83-0.92)	1.02 (0.99-1.06)	0.93 (0.86-1.01)
South East	0.81 (0.79-0.83)	0.87 (0.85-0.90)	0.88 (0.82-0.94)	0.94 (0.90-0.99)	0.94 (0.83-1.06)
East Midlands	0.95 (0.93-0.97)	0.93 (0.91-0.95)	0.99 (0.94-1.04)	1.15 (1.13-1.18)	1.11 (1.01-1.22)
South West	0.80 (0.78-0.82)	0.81 (0.79-0.83)	0.85 (0.80-0.89)	0.97 (0.94-1.00)	0.98 (0.88-1.09)
**Flu vaccination**					
Yes	0.94 (0.93-0.96)	0.85 (0.84-0.87)	0.92 (0.88-0.96)	0.90 (0.87-0.92)	0.93 (0.87-1.01)
**Count of antibiotic prescription in the one year before**	1.78 (1.76-1.80)	1.62 (1.62-1.63)	1.47 (1.45-1.48)	1.89 (1.86-1.92)	1.52 (1.49-1.56)

[1] OR, odds ratio.
[2] CI, confidence interval.
[3] BMI, Body Mass Index recorded in the last 5 years.
[4] CCI, Charlson Comorbidities Index, measured from 17 weighted conditions, including myocardial infarction, congestive heart failure, peripheral vascular disease, cerebrovascular disease, dementia, chronic pulmonary disease, Connective tissue disease, ulcer disease, mild liver disease, diabetes, hemiplegia, moderate or severe renal disease, diabetes with complications, any malignancy (including leukaemia and lymphoma), moderate or severe liver disease, metastatic solid tumour, and AIDS.
[5] IMD, Multiple Deprivation Index, quintile measured from patient-level address.
Reference group for variable sex is female, for age is 18-25, for BMI is healthy weight, for ethnicity is non-white, for CCI is very low, for smoking status is ex-smoker, for IMD is 2, for season is autumn, for region is east, for flu vaccination is no.

Table S13. Adjusted odds ratios of being prescribed an antibiotic for specific upper respiratory tract infection (URTI), cough, cold with cough, and sore throat, using data from January 2019 to March 2023, with risk factors as covariates.

	Specific URTI Adjusted OR[1] (95% CI[2])		Cough Adjusted OR[1] (95% CI[2])		Cold with cough Adjusted OR[1] (95% CI[2])		Sore throat Adjusted OR[1] (95% CI[2])	
	Incident	Prevalent	Incident	Prevalent	Incident	Prevalent	Incident	Prevalent
**Sex**								
Male	1.23 (1.22-1.25)	1.27 (1.20-1.35)	0.90 (0.89-0.90)	0.99 (0.96-1.02)	0.90 (0.90-0.91)	1.00 (0.98-1.01)	1.04 (1.03-1.05)	0.99 (0.95-1.02)
**Age**								
25-34	1.21 (1.18-1.24)	1.17 (1.00-1.36)	1.24 (1.21-1.26)	1.15 (1.06-1.24)	1.26 (1.24-1.28)	1.16 (1.10-1.22)	1.05 (1.03-1.06)	1.06 (1.01-1.12)
35-44	1.47 (1.43-1.50)	1.25 (1.07-1.45)	1.22 (1.20-1.25)	1.10 (1.02-1.18)	1.31 (1.29-1.33)	1.20 (1.14-1.26)	0.89 (0.88-0.91)	0.98 (0.94-1.04)
45-54	1.71 (1.66-1.75)	1.39 (1.20-1.60)	1.03 (1.01-1.05)	0.97 (0.91-1.04)	1.19 (1.17-1.20)	1.14 (1.08-1.19)	0.70 (0.69-0.71)	0.75 (0.71-0.80)
55-64	1.94 (1.89-1.99)	1.54 (1.32-1.79)	0.88 (0.86-0.89)	0.89 (0.83-0.95)	1.03 (1.02-1.05)	1.11 (1.06-1.17)	0.56 (0.55-0.57)	0.67 (0.62-0.72)
65-74	2.15 (2.08-2.21)	1.73 (1.50-1.99)	0.76 (0.74-0.77)	0.88 (0.82-0.95)	0.90 (0.89-0.91)	1.08 (1.03-1.14)	0.44 (0.43-0.46)	0.51 (0.46-0.56)
75+	1.93 (1.87-1.98)	1.56 (1.32-1.84)	0.78 (0.77-0.80)	0.90 (0.83-0.97)	0.95 (0.94-0.96)	1.06 (1.01-1.12)	0.34 (0.32-0.35)	0.39 (0.34-0.45)
**BMI[3]**								
Underweight	0.96 (0.92-1.01)	0.78 (0.60-1.01)	0.86 (0.84-0.88)	1.01 (0.92-1.12)	0.82 (0.81-0.84)	0.91 (0.86-0.96)	0.85 (0.82-0.88)	0.97 (0.84-1.12)
Overweight	1.06 (1.04-1.08)	1.01 (0.92-1.10)	1.13 (1.12-1.14)	1.06 (1.02-1.10)	1.15 (1.14-1.15)	1.10 (1.07-1.12)	1.09 (1.07-1.11)	1.06 (1.01-1.12)
Obese	1.09 (1.07-1.11)	1.02 (0.94-1.11)	1.21 (1.20-1.22)	1.12 (1.08-1.16)	1.23 (1.22-1.24)	1.12 (1.10-1.15)	1.11 (1.09-1.13)	1.10 (1.04-1.15)
Unknown	1.11 (1.10-1.13)	0.98 (0.90-1.07)	1.41 (1.39-1.42)	1.17 (1.13-1.21)	1.40 (1.39-1.41)	1.16 (1.13-1.19)	1.16 (1.15-1.18)	1.12 (1.06-1.17)
**Ethnicity**								
Asian	1.37 (1.34-1.41)	1.28 (1.15-1.42)	1.29 (1.27-1.32)	1.13 (1.05-1.21)	1.13 (1.12-1.15)	1.02 (0.99-1.07)	0.82 (0.80-0.84)	0.99 (0.91-1.07)
Black	1.05 (1.01-1.10)	0.81 (0.65-1.03)	0.97 (0.94-1.00)	0.81 (0.71-0.92)	0.89 (0.86-0.91)	0.81 (0.75-0.87)	0.92 (0.88-0.96)	0.92 (0.80-1.07)
Mixed	0.99 (0.94-1.05)	0.81 (0.59-1.11)	1.06 (1.01-1.11)	0.99 (0.84-1.17)	1.01 (0.98-1.04)	0.93 (0.87-1.01)	0.97 (0.92-1.02)	1.01 (0.86-1.18)
Other	0.95 (0.91-0.99)	1.09 (0.89-1.35)	0.96 (0.93-1.00)	1.09 (0.97-1.22)	0.91 (0.90-0.93)	0.96 (0.90-1.02)	0.87 (0.84-0.91)	0.98 (0.86-1.11)
Unknown	1.13 (1.11-1.15)	1.04 (0.96-1.13)	1.05 (1.04-1.06)	1.03 (1.00-1.07)	1.07 (1.06-1.08)	1.07 (1.04-1.10)	1.28 (1.26-1.30)	1.39 (1.32-1.47)
**CCI[4]**								
Low	1.01 (0.99-1.02)	0.90 (0.83-0.97)	0.97 (0.97-0.98)	1.01 (0.98-1.05)	0.99 (0.98-0.99)	0.97 (0.95-0.99)	0.84 (0.82-0.85)	0.88 (0.83-0.93)
Medium	0.89 (0.87-0.92)	0.95 (0.85-1.06)	0.96 (0.95-0.98)	1.02 (0.96-1.07)	0.93 (0.92-0.94)	0.91 (0.88-0.93)	0.77 (0.74-0.80)	0.82 (0.70-0.97)
High	0.74 (0.69-0.79)	0.71 (0.55-0.91)	0.93 (0.90-0.96)	0.99 (0.90-1.09)	0.87 (0.85-0.89)	0.81 (0.77-0.85)	0.67 (0.60-0.75)	0.65 (0.48-0.88)
Very high	0.81 (0.71-0.92)	0.61 (0.39-0.95)	0.99 (0.93-1.06)	0.83 (0.69-1.02)	0.88 (0.85-0.92)	0.75 (0.69-0.82)	0.63 (0.54-0.74)	0.99 (0.60-1.63)
**Smoking status**								
Smoker	1.15 (1.13-1.16)	1.15 (1.06-1.25)	0.88 (0.87-0.88)	1.07 (1.03-1.12)	0.88 (0.88-0.89)	1.04 (1.02-1.06)	0.98 (0.97-1.00)	0.92 (0.86-0.97)
Never smoked	1.00 (0.99-1.02)	1.04 (0.98-1.10)	1.29 (1.28-1.30)	1.06 (1.03-1.09)	1.25 (1.24-1.25)	1.03 (1.02-1.05)	1.01 (1.00-1.02)	1.02 (0.98-1.07)
Unknown	1.01 (0.95-1.08)	0.84 (0.50-1.40)	1.05 (0.97-1.12)	1.12 (0.82-1.53)	0.98 (0.93-1.02)	1.02 (0.88-1.18)	1.13 (1.10-1.17)	1.18 (1.03-1.35)
**IMD[5]**								
1 (most deprived)	0.98 (0.97-1.01)	0.88 (0.81-0.96)	1.01 (1.00-1.02)	1.01 (0.97-1.06)	1.00 (0.99-1.01)	0.96 (0.94-0.98)	0.99 (0.97-1.01)	0.96 (0.90-1.03)
3	1.01 (0.99-1.03)	1.07 (0.98-1.16)	0.96 (0.95-0.97)	1.06 (1.01-1.10)	0.94 (0.94-0.95)	0.97 (0.95-0.99)	0.98 (0.96-0.99)	0.99 (0.94-1.04)
4	1.13 (1.11-1.15)	1.10 (1.00-1.20)	0.99 (0.98-1.00)	1.08 (1.04-1.12)	0.94 (0.93-0.95)	1.00 (0.98-1.02)	0.95 (0.94-0.97)	0.97 (0.92-1.03)
5 (most affluent)	1.13 (1.11-1.15)	1.21 (1.10-1.32)	0.93 (0.92-0.94)	1.04 (1.00-1.09)	0.86 (0.85-0.87)	0.97 (0.94-0.99)	0.87 (0.86-0.89)	0.96 (0.91-1.02)

Unknown	1.10 (1.05-1.15)	1.25 (1.01-1.56)	0.91 (0.89-0.94)	1.13 (1.01-1.26)	0.89 (0.88-0.91)	0.94 (0.89-1.00)	0.88 (0.85-0.91)	0.91 (0.79-1.04)
**Season**								
Spring	0.96 (0.94-0.98)	0.96 (0.90-1.03)	0.93 (0.92-0.94)	0.92 (0.89-0.95)	0.87 (0.86-0.88)	0.92 (0.90-0.94)	1.03 (1.01-1.04)	1.11 (1.06-1.16)
Summer	1.02 (1.00-1.04)	0.95 (0.86-1.05)	0.73 (0.72-0.74)	0.76 (0.73-0.79)	0.72 (0.71-0.72)	0.80 (0.78-0.81)	1.02 (1.01-1.04)	1.05 (0.99-1.12)
Winter	0.97 (0.95-0.98)	1.02 (0.94-1.10)	1.18 (1.17-1.19)	1.22 (1.18-1.26)	1.16 (1.15-1.17)	1.14 (1.12-1.16)	1.07 (1.05-1.08)	1.21 (1.15-1.27)
**Region**								
London	1.00 (0.98-1.02)	0.75 (0.65-0.88)	1.25 (1.23-1.28)	0.91 (0.84-0.98)	0.89 (0.88-0.90)	0.81 (0.77-0.84)	0.98 (0.95-1.00)	0.84 (0.77-0.91)
North East	0.67 (0.65-0.69)	0.81 (0.69-0.96)	0.72 (0.71-0.74)	0.85 (0.80-0.90)	0.79 (0.78-0.80)	0.87 (0.84-0.89)	0.87 (0.85-0.89)	0.84 (0.76-0.92)
North West	0.61 (0.59-0.62)	0.78 (0.70-0.87)	0.78 (0.77-0.79)	0.85 (0.81-0.88)	0.90 (0.89-0.91)	0.95 (0.93-0.97)	0.87 (0.85-0.89)	0.93 (0.88-0.99)
West Midlands	0.94 (0.92-0.97)	0.78 (0.69-0.89)	1.17 (1.15-1.20)	0.94 (0.87-1.01)	1.23 (1.21-1.25)	1.04 (1.01-1.08)	0.96 (0.93-0.99)	0.90 (0.82-0.99)
Yorkshire and The Humber	0.91 (0.89-0.92)	0.70 (0.64-0.77)	0.85 (0.84-0.86)	0.82 (0.78-0.86)	1.04 (1.03-1.05)	0.97 (0.95-0.99)	1.00 (0.98-1.02)	0.83 (0.79-0.88)
South East	0.60 (0.58-0.62)	0.82 (0.71-0.95)	0.92 (0.90-0.93)	1.02 (0.96-1.08)	0.89 (0.88-0.90)	0.93 (0.90-0.96)	0.88 (0.85-0.90)	0.81 (0.75-0.88)
East Midlands	0.68 (0.66-0.69)	0.87 (0.79-0.95)	0.89 (0.88-0.90)	0.91 (0.88-0.95)	1.02 (1.01-1.03)	1.05 (1.02-1.07)	0.97 (0.96-0.99)	0.93 (0.89-0.98)
South West	0.53 (0.52-0.54)	0.60 (0.53-0.69)	0.76 (0.75-0.77)	0.88 (0.84-0.92)	0.78 (0.77-0.78)	0.87 (0.84-0.89)	0.88 (0.86-0.90)	0.77 (0.72-0.82)
**Flu vaccination**								
Yes	0.95 (0.94-0.97)	0.95 (0.88-1.02)	0.81 (0.80-0.82)	0.96 (0.93-0.99)	0.83 (0.83-0.84)	0.97 (0.95-0.99)	0.86 (0.85-0.88)	0.97 (0.92-1.02)
**Count of antibiotic prescription in the one year before**	1.84 (1.83-1.86)	1.51 (1.48-1.54)	1.28 (1.28-1.28)	1.29 (1.28-1.30)	1.32 (1.32-1.33)	1.28 (1.28-1.29)	2.30 (2.28-2.32)	1.68 (1.66-1.70)

[1] OR, odds ratio.
[2] CI, confidence interval.
[3] BMI, Body Mass Index recorded in the last 5 years.
[4] CCI, Charlson Comorbidities Index, measured from 17 weighted conditions, including myocardial infarction, congestive heart failure, peripheral vascular disease, cerebrovascular disease, dementia, chronic pulmonary disease, Connective tissue disease, ulcer disease, mild liver disease, diabetes, hemiplegia, moderate or severe renal disease, diabetes with complications, any malignancy (including leukaemia and lymphoma), moderate or severe liver disease, metastatic solid tumour, and AIDS.
[5] IMD, Multiple Deprivation Index, quintile measured from patient-level address.
Reference group for variable sex is female, for age is 18-25, for BMI is healthy weight, for ethnicity is non-white, for CCI is very low, for smoking status is ex-smoker, for IMD is 2, for season is autumn, for region is east, for flu vaccination is no.

Supplementary Figures

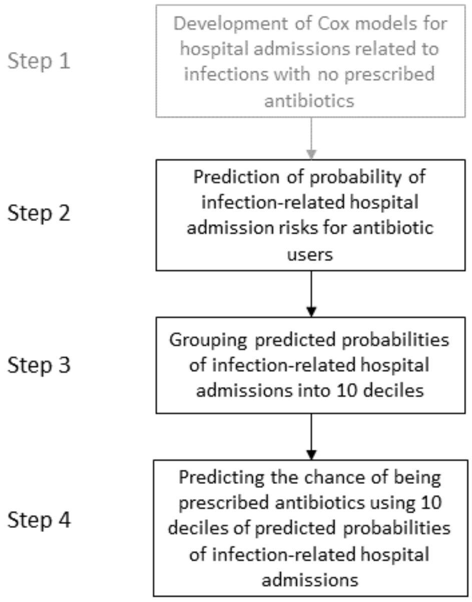

Figure S1. Flowchart of analyses.

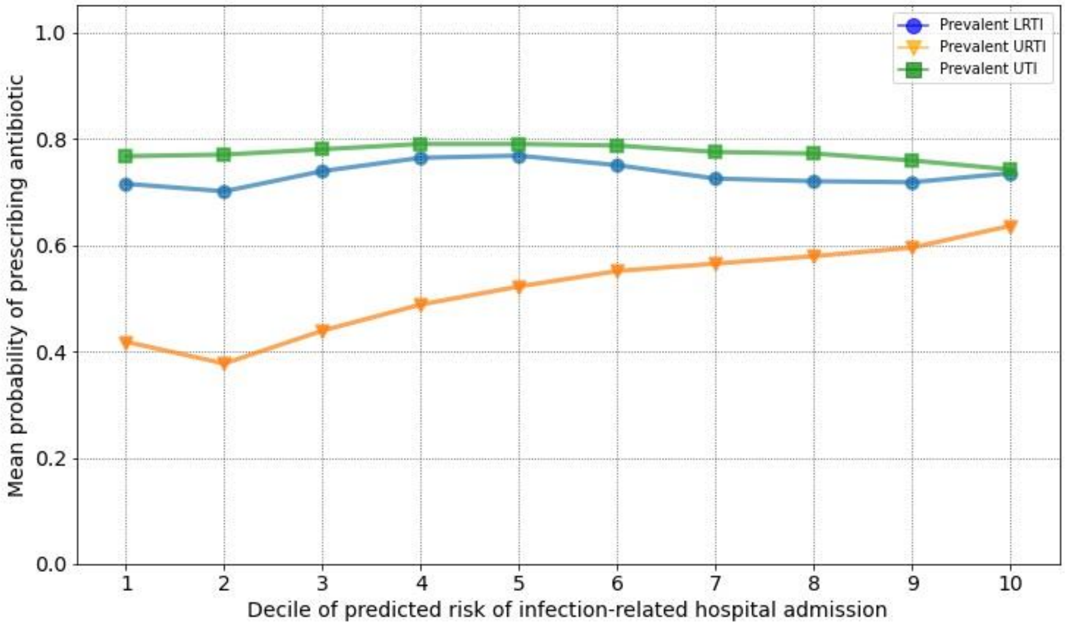

Figure S2. Probability of antibiotic prescribing by predicted risk level of hospital admission related to prevalent lower respiratory tract infection (LRTI), upper respiratory tract infection (URTI), and urinary tract infection (UTI), where x axis shows deciles of predicted risk of infection-related hospital admissions and y axis shows the mean probability of antibiotic prescribing.

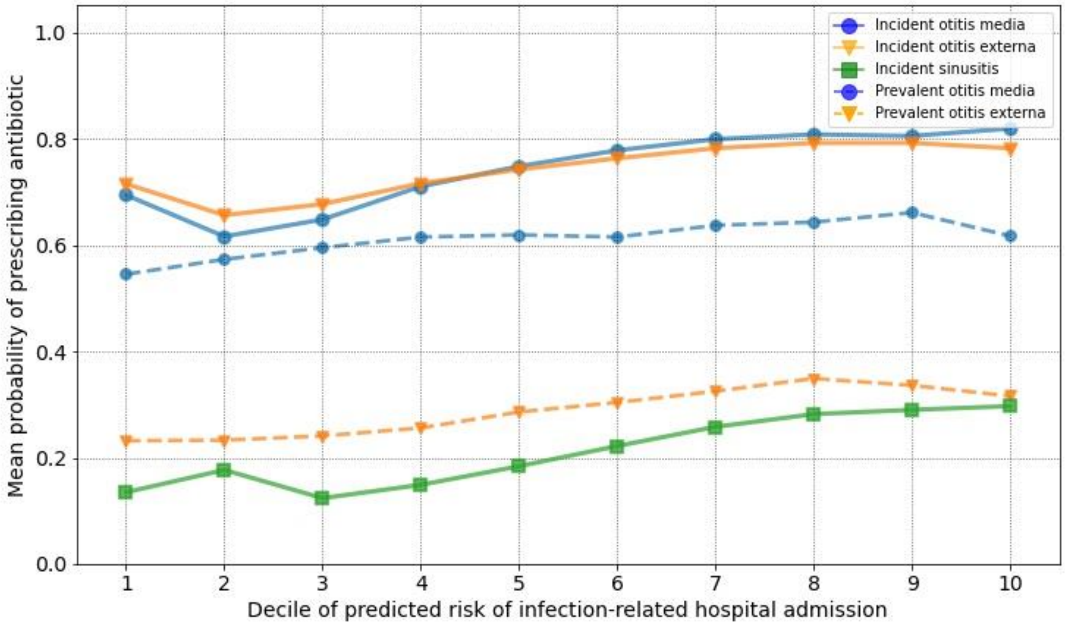

Figure S3. Probability of antibiotic prescribing by predicted risk level of hospital admission related to incident and prevalent otitis media, otitis externa, and sinusitis (except prevalent sinusitis), where x axis shows deciles of predicted risk of infection-related hospital admissions and y axis shows the mean probability of antibiotic prescribing.

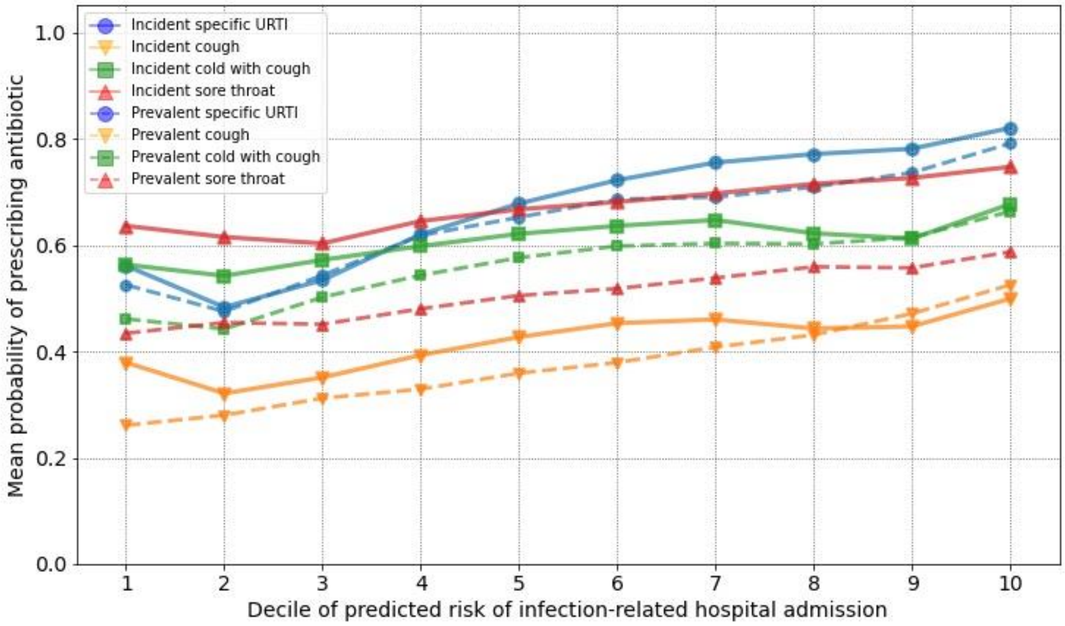

Figure S4. Probability of antibiotic prescribing by predicted risk level of hospital admission related to incident and prevalent upper respiratory tract infections (URTIs), including specific URTI, cough, cold with cough, and sore throat, where x axis shows deciles of predicted risk of infection-related hospital admissions and y axis shows the mean probability of antibiotic prescribing.

TRIPOD checklist

Table S14. TRIPOD checklist.

Section/Topic			Checklist Item	Page
**Title and abstract**				
Title	1	D;V	Identify the study as developing and/or validating a multivariable prediction model, the target population, and the outcome to be predicted.	1
Abstract	2	D;V	Provide a summary of objectives, study design, setting, participants, sample size, predictors, outcome, statistical analysis, results, and conclusions.	1
**Introduction**				
Background and objectives	3a	D;V	Explain the medical context (including whether diagnostic or prognostic) and rationale for developing or validating the multivariable prediction model, including references to existing models.	2
	3b	D;V	Specify the objectives, including whether the study describes the development or validation of the model or both.	2
**Methods**				
Source of data	4a	D;V	Describe the study design or source of data (e.g., randomized trial, cohort, or registry data), separately for the development and validation data sets, if applicable.	2-3
	4b	D;V	Specify the key study dates, including start of accrual; end of accrual; and, if applicable, end of follow-up.	2-3
Participants	5a	D;V	Specify key elements of the study setting (e.g., primary care, secondary care, general population) including number and location of centres.	2
	5b	D;V	Describe eligibility criteria for participants.	2
	5c	D;V	Give details of treatments received, if relevant.	2-3
Outcome	6a	D;V	Clearly define the outcome that is predicted by the prediction model, including how and when assessed.	3
	6b	D;V	Report any actions to blind assessment of the outcome to be predicted.	-
Predictors	7a	D;V	Clearly define all predictors used in developing or validating the multivariable prediction model, including how and when they were measured.	3
	7b	D;V	Report any actions to blind assessment of predictors for the outcome and other predictors.	-
Sample size	8	D;V	Explain how the study size was arrived at.	-
Missing data	9	D;V	Describe how missing data were handled (e.g., complete-case analysis, single imputation, multiple imputation) with details of any imputation method.	3
Statistical analysis methods	10a	D	Describe how predictors were handled in the analyses.	3
	10b	D	Specify type of model, all model-building procedures (including any predictor selection), and method for internal validation.	3-4
	10c	V	For validation, describe how the predictions were calculated.	3
	10d	D;V	Specify all measures used to assess model performance and, if relevant, to compare multiple models.	4
	10e	V	Describe any model updating (e.g., recalibration) arising from the validation, if done.	-
Risk groups	11	D;V	Provide details on how risk groups were created, if done.	3
Development vs. validation	12	V	For validation, identify any differences from the development data in setting, eligibility criteria, outcome, and predictors.	-
**Results**				
Participants	13a	D;V	Describe the flow of participants through the study, including the number of participants with and without the outcome and, if applicable, a summary of the follow-up time. A diagram may be helpful.	4
	13b	D;V	Describe the characteristics of the participants (basic demographics, clinical features, available predictors), including the number of participants with missing data for predictors and outcome.	4
	13c	V	For validation, show a comparison with the development data of the distribution of important variables (demographics, predictors and outcome).	-
Model development	14a	D	Specify the number of participants and outcome events in each analysis.	4
	14b	D	If done, report the unadjusted association between each candidate predictor and outcome.	-
Model specification	15a	D	Present the full prediction model to allow predictions for individuals (i.e., all regression coefficients, and model intercept or baseline survival at a given time point).	4-5
	15b	D	Explain how to the use the prediction model.	4-5
Model performance	16	D;V	Report performance measures (with CIs) for the prediction model.	4
Model-updating	17	V	If done, report the results from any model updating (i.e., model specification, model performance).	-
**Discussion**				
Limitations	18	D;V	Discuss any limitations of the study (such as nonrepresentative sample, few events per predictor, missing data).	5-6
Interpretation	19a	V	For validation, discuss the results with reference to performance in the development data, and any other validation data.	5-7

	19b	D;V	Give an overall interpretation of the results, considering objectives, limitations, results from similar studies, and other relevant evidence.	5-7
Implications	20	D;V	Discuss the potential clinical use of the model and implications for future research.	7
**Other information**				
Supplementary information	21	D;V	Provide information about the availability of supplementary resources, such as study protocol, Web calculator, and data sets.	4-5
Funding	22	D;V	Give the source of funding and the role of the funders for the present study.	8-9

*Items relevant only to the development of a prediction model are denoted by D, items relating solely to a validation of a prediction model are denoted by V, and items relating to both are denoted D;V. We recommend using the TRIPOD Checklist in conjunction with the TRIPOD Explanation and Elaboration document.
